# Supplementary material for: Live-cell quantitative monitoring reveals distinct, high-affinity Gβγ regulations of GIRK2 and GIRK1/2 channels
Source: Nat Commun. 2025 Nov 24;16:11607. doi: 10.1038/s41467-025-66730-8 (PMC12748987; doi:10.1038/s41467-025-66730-8)
Supplement: Supplementary file 1 — Supplementary Information [file 41467_2025_66730_MOESM1_ESM.pdf]

Supplementary Material

Live-cell quantitative monitoring reveals distinct, high-affinity Gβγ regulations of GIRK2 and GIRK1/2 channels.

Reem Handklo-Jamal[1], Tal Keren Raifman[1], Boris Shalomov[1], Patrick Hofer[2], Uri Kahanovitch[1], Theres Friesacher[2], Galit Tabak[1], Vladimir Tsemakhovich[1], Haritha P. Reddy[1, ¶], Orna Chomsky-Hecht[3], Debi Ranjan Tripathy[1,3], Kerstin Zuhlke[4], Carmen W. Dessauer[5], Enno Klussmann[4,6], Yoni Haitin[1,7], Joel A. Hirsch[3,7], Anna Stary-Weinzinger[2*], Daniel Yakubovich[8*], Nathan Dascal[1,7*]

[1] Department of Physiology and Pharmacology, Faculty of Health and Medical Sciences, Tel Aviv University, Tel Aviv, Israel

[2] Department of Pharmaceutical Sciences, Division of Pharmacology and Toxicology, University of Vienna, Josef-Holaubek-Platz 2, 1090 Vienna, Austria.

[3] Department of Biochemistry & Molecular Biology, School of Neurobiology, Biochemistry and Biophysics, George S. Weiss Faculty of Life Sciences, Tel Aviv University, Tel Aviv, Israel

[4] Max-Delbrück-Center for Molecular Medicine in the Helmholtz Association (MDC), Berlin, Germany

[5] Department of Integrative Biology and Pharmacology, University of Texas Health Science Center, Houston, Texas, USA

[6] DZHK (German Centre for Cardiovascular Research), partner site Berlin, Germany

[7] Sagol School of Neuroscience, Tel Aviv University, Tel Aviv, Israel

[8] The Adelson School of Medicine, Ariel University, Ariel 4077625, Israel

¶ Present address: Department of Neuroscience, Faculty of Health and Medical Sciences, University of Copenhagen, Blegdamsvej 3B, 2200 Copenhagen N, Denmark

* Correspondence to: Nathan Dascal dascaln@tauex.tau.ac.il, Daniel Yakubovich danielya@ariel.ac.il, Anna Stary-Weinzinger anna.stary@univie.ac.at

Supplementary Methods

Modeling GIRK1/2 basal activity and activation by expression of Gβγ with four kinetic models

To describe GIRK1/2 activation by Gβγ with the inclusion of a mechanistic explanation of I_{basal}, we utilized four gating models (Supplementary Fig. 8a): #1, concerted activation, non-cooperative binding; #2, concerted activation, cooperative binding; #3, graded contribution, non-cooperative binding; and #4, graded contribution, cooperative binding model. Concerted activation models are based on the assumption of four Gβγ required for channel opening. The concerted activation, cooperative binding model #2 is the modified WTM model used throughout this paper. Graded contribution models are based on increasing contribution to $P_{o,max}$ of each sequential Gβγ-bound state. We have previously described the graded contribution non-cooperative binding model[1], and the graded contribution cooperative binding model was described in detail by Berlin et al.[2].

Since the basal activity of GIRK12 is highly Gβγ-dependent, and this phenomenon was shown to be dependent on differential recruitment of Gα and Gβγ to the GIRK1/2 microenvironment[3], we first utilized each of the four models of Supplementary Fig. 8a to estimate the basal endogenous Gα and Gβγ available for channel's gating ($G\alpha_{endo}$ and $G\beta\gamma_{endo}$, where endo stays for endogenous). The basal available Gβγ and Gα were calculated from the experimentally observed I_{basal} as described previously[1, 2]. For simulation, we constructed systems of differential equations based on these schemes and solved them numerically in Berkley Madonna (Berkeley Madonna, Inc., Albany, CA)[4] utilizing 4th order Runge-Kutta integration method. The simulations were run till apparent steady state was achieved.

For calculations we used the following parameters: $P_{o,max}$ (open probability value, observed with 5 ng Gβ RNA and 1-2 Gγ RNA) ~ 0.105[1], P_o in the absence of Gβγ ~0.00273 (assuming 10% Gβγ-independent activity out of total I_{basal} of GIRK1/2 , $P_{o,basal}$ = 0.0273 (open probability in absence of Gβγ expression, c=26 % of maximal open probability; Fig. 4F) and Po, agonist = 0.0525 (open probability corresponding to full endogenous Gβγ dissociation from Gα induced by agonist, which is 50% of that of $P_{o,max}$ with 5 ng Gβ RNA[1]. Utilizing the $P_{o,max}$ and single channel current, we estimated channel density to be 13.7 channels/µm^2. The above described data were substituted to system of equations relevant for each model as described[1, 2] and solved in Matlab 6.5 for Windows. This procedure was conducted for a range of K_d values, thus generating initial values matrix containing [$K_{d,}$ $G\beta\gamma_{endo}$, $G\alpha_{endo}$]. Estimated values are shown in Supplementary Fig. 8b. Each [$K_{d,}$ $G\beta\gamma_{endp}$, $G\alpha_{endo}$] was subsequently utilized for simulation of a complete dose response to expressed Gβγ. In order to make calculations more time-efficient, the dose response was simulated as a steady-state solution of a system of differential equations. Gating schemes described in Supplementary Fig. 8a were combined with agonist-independent G-protein dissociation reaction:

$$(1) \qquad G\alpha_{GDP} + G\beta\gamma \underset{k_{off,G}}{\overset{k_{on,G}}{\rightleftarrows}} G\alpha_{GDP}G\beta\gamma$$

where $G\alpha_{endo}=G\alpha_{GDP}+G\alpha_{GDP}G\beta\gamma$ and $G\beta\gamma_{endo}=G\beta\gamma+G\alpha_{GDP}G\beta\gamma$, and $G\alpha_{endo}$ and $G\beta\gamma_{endo}$ are calculated values obtained for each K_d value as described above.

Simulation of models based on cooperative binding of Gβγ to the channel was based on solution of the following differential equations system:

(2) $dG\alpha_{GDP}/dt=k_{off,G}\cdot G\alpha_{GDP}G\beta\gamma-k_{on,G}\cdot G\alpha_{GDP}\cdot G\beta\gamma$

(3) $dG\beta\gamma/dt= k_{off,G}\cdot G\alpha_{GDP}G\beta\gamma+k_{off}\cdot(C_1+2\cdot\mu\cdot C_2+3\cdot\mu^2\cdot C_3+4\cdot\mu^3\cdot C_4)-$

$- G\beta\gamma\cdot(k_{on,G}\cdot G\alpha_{GDP}+4\cdot k_{on}\cdot C_0+3\cdot k_{on}\cdot C_1+2\cdot k_{on}\cdot C_2+ k_{on}\cdot C_3)$

(4) $dG\alpha_{GDP}G\beta\gamma/dt= k_{on,G}\cdot G\alpha_{GDP}\cdot G\beta\gamma- k_{off,G}\cdot G\alpha_{GDP}\cdot G\beta\gamma$

(5) $dC_0/dt= -4\cdot k_{on}\cdot G\beta\gamma\cdot C_0+k_{off}\cdot C_1$

(6) $dC_1/dt=4\cdot k_{on}\cdot G\beta\gamma\cdot C_0-3\cdot k_{on}\cdot G\beta\gamma\cdot C_1- k_{off}\cdot C_1+ 2\cdot\mu\cdot k_{off}\cdot C_2$

(7) $dC_2/dt=3\cdot k_{on}\cdot G\beta\gamma\cdot C_1-2\cdot k_{on}\cdot G\beta\gamma\cdot C_2- 2\cdot\mu\cdot k_{off}\cdot C_2+3\cdot\mu^2\cdot k_{off}\cdot C_3$

(8) $dC_3/dt=2\cdot k_{on}\cdot G\beta\gamma\cdot C_2-1\cdot k_{on}\cdot G\beta\gamma\cdot C_3- 3\cdot\mu^2\cdot k_{off}\cdot C_3+4\cdot\mu^3\cdot k_{off}\cdot C_4$

(9) $dC_4/dt=1\cdot k_{on}\cdot G\beta\gamma\cdot C_3- 4\cdot\mu^3\cdot k_{off}\cdot C_4$

Simulation for model based on non-cooperative binding of Gβγ to channel was based on solution of the following differential equations system:

(10) $dG\alpha_{GDP}/dt=k_{off,G}\cdot G\alpha_{GDP}G\beta\gamma-k_{on,G}\cdot G\alpha_{GDP}\cdot G\beta\gamma$

(11) $dG\beta\gamma/dt= k_{off,G}\cdot G\alpha_{GDP}G\beta\gamma +k_{off}\cdot(C_1+2\cdot\mu\cdot C_2+3\cdot\mu^2\cdot C_3+4\cdot\mu^3\cdot C_4)-$

$-G\beta\gamma\cdot(k_{on,G}\cdot G\alpha_{GDP}+4\cdot k_{on}\cdot C_0+3\cdot k_{on}\cdot C_1+2\cdot k_{on}\cdot C_2+ k_{on}\cdot C_3)$

(12) $dG\alpha_{GDP}G\beta\gamma/dt= k_{on,G}\cdot G\alpha_{GDP}G\beta\gamma- k_{off,G}\cdot G\alpha_{GDP}G\beta\gamma$

(13) $dC_0/dt= -4\cdot k_{on}\cdot G\beta\gamma\cdot C_0+k_{off}\cdot C_1$

(14) $dC_1/dt=4\cdot k_{on}\cdot G\beta\gamma\cdot C_0-3\cdot k_{on}\cdot G\beta\gamma\cdot C_1- k_{off}\cdot C_1+ 2\cdot k_{off}\cdot C_2$

(15) $dC_2/dt=3\cdot k_{on}\cdot G\beta\gamma\cdot C_1-2\cdot k_{on}\cdot G\beta\gamma\cdot C_2- 2\cdot k_{off}\cdot C_2+3\cdot k_{off}\cdot C_3$

(16) $dC_3/dt=2\cdot k_{on}\cdot G\beta\gamma\cdot C_2-1\cdot k_{on}\cdot G\beta\gamma\cdot C_3- 3\cdot k_{off}\cdot C_3+4\cdot k_{off}\cdot C_4$

(17) $dC_4/dt=1\cdot k_{on}\cdot G\beta\gamma\cdot C_3- 4\cdot k_{off}\cdot C_4$

For models based on graded contribution of each Gβγ occupied state to channel activity the open probability was calculated according to:

(18) $$P_o = P_{o,max}\cdot\sum_1^4 \varphi_i\cdot\frac{C_i}{C_{total}}$$

where φ_i is the contribution factor of each occupied Gβγ state to open probability, and C_{total} is channel concentration ($C_{total}=\sum_{i=0}^{i=4} C_i$). φ_i values were adopted from Yakubovich et al.[1] and Berlin et al.[2] and based on data published by Ivanova-Nikolova et al. and Sadja et al.[5, 6].

For models based on concerted gating the open probability was calculated according to:

(19) $$P_o = P_{o,max}\cdot\frac{C_4}{C_{total}}$$

These models are based on the same assumption as used in WTM model, i.e. only 4 Gβγ-occupied channel is available for opening.

In all models C_0-C_4 correspond to 0-4 Gβγ occupied state of the channel, k_{on} =1e7 $M^{-1}s^{-1}$ (similar to value utilized by Berlin at al. and in agreement with the diffusion limit[2, 7], k_{off} =K_d/k_{on}, μ is the cooperativity factor of Gβγ binding to each consecutive Gβγ occupied state[7]. $k_{on,G}$ =0.7e6 $M^{-1}s^{-1}$ and $k_{off,G}$ =0.0013 s^{-1} as reported by Sarvazyan et al.[8]. For simulation of response to expressed Gβγ initial values of [K_d $G\alpha_{endo}$, $G\beta\gamma_{endo}$+$G\beta\gamma_{expressed}$] were utilized for each consequtive run of differential equation system solution, thus generating matrix of [$G\beta\gamma_{expressed}$ P_o] values for each K_d. Differential equations systems were solved in Berkeley Madonna for Windows utilizing 4th order Runge-Kutta integration method. All systems were allowed to reach steady-state. The results of simulation were compared to experimental dose-response curves. For selection of optimal [K_d $G\alpha_{endo}$ $G\beta\gamma_{endo}$] values we utilized two criteria: a) the stability of G-protein concentration estimation as seen from Supplementary Fig. 8b – i.e. an optimal model is expected to generate stable estimation of G protein concentration over a wide range of tested K_d values, and b)

resemblance to superimposed dose-response by visual inspection. Simulations of all models are shown in Fig. 4g and Supplementary Fig. 8d,e.

<u>Gβγ expression and purification</u>

The pFastBac Dual vector (Invitrogen) was utilized to coexpress the bovine $G\gamma_2$ (WT and C68S) and $G\beta_1$ genes in Sf9 insect cells. The bovine Gγ2 gene was subcloned downstream of a His-tag and a Tobacco Etch Virus (TEV) protease recognition site in the first multiple cloning site, under the control of the polyhedrin promoter. The Gβ1 gene was inserted into the second MCS, under the control of the p10 promoter.

His_6-Gβγ and His_6-$G\beta\gamma_{C68S}$ were purified essentially as described[9]. The pFastBac construct was transformed into DH10Bac competent *E. coli* to generate a recombinant bacmid. The recombinant bacmid was isolated and used for transfection to SF9 cells in presence of CellFectin II reagent (ThermoFisher Scientific) to generate recombinant baculovirus. Further, this baculovirus stock was amplified and optimized for maximum protein expression via infection to *Trichoplusia ni* (T.ni) cells. Infected T.ni cells were grown for 60-72 hours, harvested by centrifugation at 200×g and stored at -80°C until further use.

For His-$G\beta\gamma_{C68S}$ purification, cells were suspended and homogenized using glass homogenizer in 20 mM Hepes pH 8.0, 100 mM NaCl, 3 mM $MgCl_2$, 100 mM EDTA, 10 mM β-mercaptoethanol, cocktail protease inhibitors (PIC), 1% Triton X-100. Then the cells were lysed in sonicator using a program 5/25 sec on/off pulse for 30 min and subjected to centrifugation at 200,000×g for 45 min. The soluble fraction was filtered using 0.4 µM filter and His-tag $G\beta\gamma_{C68S}$ was purified by sequential Ni^{2+} chelate and size-exclusion [Superdex-75 HiPrep (GE Healthcare)] column chromatography. Final buffer conditions were: 20 mM Hepes pH 8.0, 100 mM NaCl, 1 mM $MgCl_2$, 10 mM β-mercaptoethanol.

For His-$G\beta\gamma_{WT}$ purification, cells were resuspended in lysis buffer (20 mM HEPES, pH 8.0; 150 mM NaCl; 5 mM $MgCl_2$; 1 mM EDTA; 1 mM EGTA; 10 mM β-mercaptoethanol; PIC), homogenized, and lysed by sonication (5 cycles of 10 s ON/90 s OFF). The lysate was first centrifuged at low speed, and then the supernatant was centrifuged at 200,000×g for 45 min at 4 °C (Ti-70 rotor). The resulting membrane pellet was collected and stored at -80 °C. Later, membranes were thawed, resuspended in wash buffer (20 mM Hepes pH 8, 100 mM NaCl, 2mM $MgCl_2$, 10 mM β-mercaptoethanol) and homogenized. Membrane proteins were extracted with 1% sodium cholate at 4 °C, and the extract was clarified by centrifugation at 200,000×g for 30 min. The supernatant was supplemented with 10 mM imidazole, 0.5 mM C12E10, 0.01 mM GDP and PIC, and applied to 1 ml Ni-NTA column (Protein ARK, Rotherham, UK). Endogenous Gα was removed by elution with Gα elution buffer (20 mM HEPES, pH 8.0; 10 mM β-mercaptoethanol; 100 mM $MgCl_2$; 100 mM NaCl; 15 mM imidazole; 0.05 mM GDP; 1% cholate; 0.03 mM $AlCl_3$; 10 mM NaF) at 33 °C. Gβγ subunits were subsequently eluted with Gβγ elution buffer (20 mM HEPES, pH 8.0; 10 mM β-mercaptoethanol; 100 mM NaCl; 0.01 mM GDP; 1% cholate; 500 mM imidazole) at 4 °C. Gβγ fractions were applied to a HiTrap Capto Q column (Cytiva, Marlborough, MA, USA). Proteins were washed and eluted using a NaCl gradient, and fractions containing pure Gβγ were pooled, concentrated, and exchanged into storage buffer (20 mM HEPES, pH 8.0; 3 mM DTT; 50 mM NaCl; 11.4 mM CHAPS). The fraction purity was analyzed using SDS-PAGE. The protein was also characterized by Western blot using anti-$G\beta_1$ (GTX114442) and anti-His tag (Roche 11 965 085 001) antibody (Supplementary Table 10).

Extracellular HA staining

Extracellular HA staining of the oocytes was performed as described[10]. Briefly, oocytes were fixated with 4% formaldehyde for 30 min, blocked in 5% milk in Ca^{2+}-free ND96 solution for 1 hour, incubated with mouse anti-HA antibody (1:333; Santa Cruz Biotechnology, Dallas, TX, USA) in 2.5% milk-Ca^{2+}-free ND96 for 1 hour, washed thrice, and incubated with DyLight405-conjugated anti-mouse antibody (KPL, Gaithersburg, MD, USA). Oocytes were then kept at 4°C in the dark in Ca^{2+}-free ND96 solution until imaged.

Pull-down assays, autoradiograms and western blots.

Pull-down was done with *in vitro* translated (*ivt*) [^{35}S]methionine-labelled proteins prepared in rabbit reticulocyte lysate (Promega, Madison, WI, USA), or unlabeled proteins from whole-oocyte lysates. Lysates were mixed with ~2 µg of either purified His-$G\beta\gamma_{WT}$ or purified His-$G\beta\gamma_{C68S}$ in 300 µl of the incubation buffer (in mM: 150 KCl, 50 Tris, 0.6 $MgCl_2$, 1 EDTA, 0.1% Lubrol or 0.5% CHAPS; pH 7.4). 1/60 of the mixture before the pull-down was loaded, usually on a separate gel, as 'input'. The reaction mixture was incubated while shaking for 60 min at room temperature, then 30 µl beads and imidazole (10 mM final concentration) were added, and incubated for 30 min at 4°C. His-Gβγ was pulled-down using HisPur^{TM} Ni-NTA Resin affinity beads (ThermoFisher Scientific, Rockford, IL, USA). The beads were washed three times with 500 µl buffer containing 15 mM imidazole. Elution was done with 30 µl incubation buffer supplemented with 250 mM imidazole. Eluates were electrophoresed on 12% polyacrylamide-SDS gel in running buffer (final concentrations: 2% SDS, 10% glycerol, 5% β-mercaptoethanol, 0.05% Bromophenol Blue, 62.5 mM Tris-HCl pH 6.8). For autoradiography ([^{35}S]methionine), gels were dried and imaged using Sapphire™ Biomolecular Imager (Azure Biosystems, Dublin, CA, USA). Autoradiograms were analyzed using ImageQuant 5.2 (GE Healthcare) and ImageJ/Fuji. Binding was calculated as percentage or fraction of the input of this construct in the same experiment. Signal in each band was assessed as (mean intensity)×(area) using ImageJ/Fiji (https://imagej.net/software/fiji/). To quantify the expressed Gβ, endogenous Gβ signal from oocytes expressing the channel alone was subtracted from the total signal.

For WB, plasma membranes were solubilized in 35 µl running buffer and heated to 65°C for 5 min. Whole oocyte lysates or pull-down eluates were supplemented with 4× or 6× running buffer to 1× final concentration. Samples were electrophoresed as above and transferred to nitrocellulose membranes for WB. (For qWB of Gβ, known amounts of purified His-Gβγ were run on the same gel for the construction of the calibration curve). Gβ was detected with anti-GNB1 antibody (GTX114442) at 1:500 or 1:1000 dilution on Fusion FX7 (Witec AG, Sursee, Switzerland). YFP was detected with the anti-GFP antibody (Abcam ab6556), 1:500 – 1:1000. For both PMs and whole oocytes, endogenous Gβ signal was subtracted as for autoradiograms.

Confocal imaging

Confocal imaging and analysis were performed as described[11], with Zeiss 510, Ziess 710 or Leica TCS SP5 confocal microscopes, using a 20× objective. Live oocytes were images at their animal hemisphere in ND96 or NDE solutions. Giant membrane patches were imaged at their edges, so both the membrane and the background were visible. Images were acquired using spectral (λ)-mode or channel mode. For imaging the following wavelength parameters were used: YFP, excitation 514 nm, imaging at 525–540 nm; DyLight 650, excitation 633 nm, imaging at 663-673 nm. For whole oocytes, fluorescence signals at the maximum emission wavelength are

averaged from three regions of interest using Zeiss LSM Image Browser (Carl Zeiss Jena GmbH), ImageJ or LAS AF (Lecia Microsystems CMC GmbH) Image software. For giant membrane patches, the entire visible membrane and background were averaged. Averaged background for each signal, and the average net signal from uninjected (native) oocytes of the same experiment, were subtracted to obtain net signal. For the effect of Gβγ on the expression of GIRK2 and GIRK2HA, signals were normalized by dividing the signal of each oocyte with the average of the group that did not contain Gβγ.

Molecular dynamics simulations

Primary structures of G1NC and G2NC were generated by fusing the NT and CT of human GIRK1 (UniProt-ID: P48549; a.a.1-84 and 183-501) and human GIRK2 (UniProt-ID: P48051, a.a. 1-93 and 193-414), respectively (Fig. 8). The heatmaps in Fig. 8 show G412 as the last a.a., which corresponds to G414 of mGIRK2 (Supplementary Fig. 9). Additionally, Gβγ (UniProt-ID: P62873, P59768) units were incorporated into the sequences. Truncated G1NC ($G1NC_{\Delta dCT}$) and G1NC ($G2NC_{trunc}$) were obtained by omitting a.a. 379-501 and a.a. 1-52 and 381-412 for G1NC and G2NC, respectively (same as $G1NC_{\Delta dCT}$ and $G2NC_{trunc}$ used in biochemical experiments, Fig. 1e). Heterotetramers bound to four Gβγ were modeled using AlphaFold 3[12]. Coarse-grained constructs were modeled with CHARMM-GUI (assessed July 2024)[13]. The G1NC membrane system was constructed using the AlphaFold-predicted structure of G1NC, which was converted to a coarse-grained representation with the CHARMM-GUI Bilayer Builder (details in Supplementary Table 11) and embedded in a POPC (1-Palmitoyl-2-oleoyl-sn-glycero-3-phosphocholine) membrane using the Martini bilayer builder of CHARMM-GUI. The non-prenylated Gβγ subunits were replaced with prenylated variants, and two additional Gβγ subunits were positioned in the cytosol using PyMOL. The system was subsequently solvated, minimized, and equilibrated following standard CHARMM-GUI protocols. Simulations were conducted with Gromacs 2022.3[14] based on the Martini force field Elnedyn22p[15] and polarizable water[16] in 100mM KCl. For the geranylgeranyl moiety, previously published parameters were used[17]. Minimization and equilibration procedures followed the CHARMM-GUI protocol. Simulation runs of 1 and 5 µs each were performed with a time step of 20 fs. During the production runs, the temperature was set to 310 K using the v-rescale thermostat[18], and the pressure was kept at 1 bar with the Parrinello-Rahman barostat[19]. Lennard-Jones potentials and Coulombic interactions are switched off between 9 to 12 Å and 0 to 12 Å, respectively. Equilibration of the simulations was assessed by comparing G1NC–prenylation tail interactions over 1 µs and 5 µs runs, which showed consistent and stable binding (Supplementary Figs. 10c). Contacts were analyzed using MDAnalysis[20] with a distance cut-off of 6 Å. For atomistic simulations, a structure was extracted from a coarse-grained G1NC simulation at 1 µs and converted to an all-atom representation using the CHARMM-GUI Martini to All-atom converter[21] with the Amber14sb force field[22]. The system was solvated with SPC water and 108 mmol K+ and Cl− ions. Prenylated Gβγ subunits from previous simulations were included. Minimization, equilibration, and three production runs of 500 ns each were performed with a time step of 2 fs following the CHARMM-GUI protocol.

Supplementary Figures

a

	$I_{\beta\gamma}$, µA	I_{basal}, nA			R_a			$R_{\beta\gamma}$			N
		mean	SEM	n	mean	SEM	n	mean	SEM	n	
GIRK2	2-4	64.2	7.1	49	8.2	0.8	49	67.3	7.9	52	7
	>4	137.7	19.2	48	5.1	0.4	48	68.9	9.3	31	5
GIRK2HA	3-10	69.8	16.4	16	5.7	0.7	16	78.0	9.6	21	3
	>10	348.7	34.0	23	4.9	0.7	23	49.4	7.1	18	3

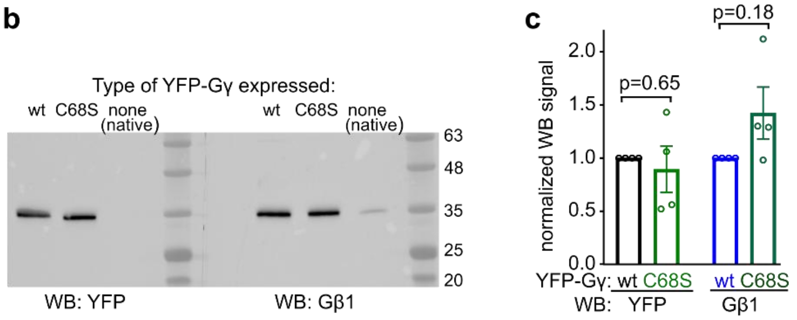

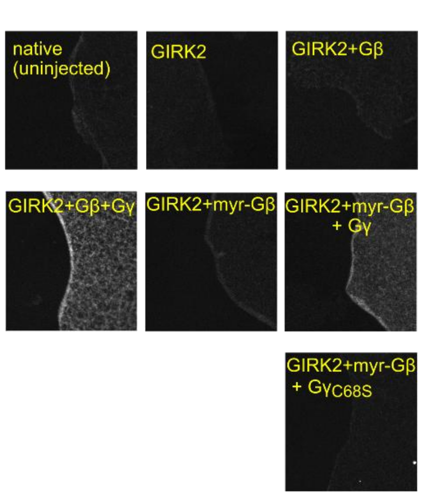

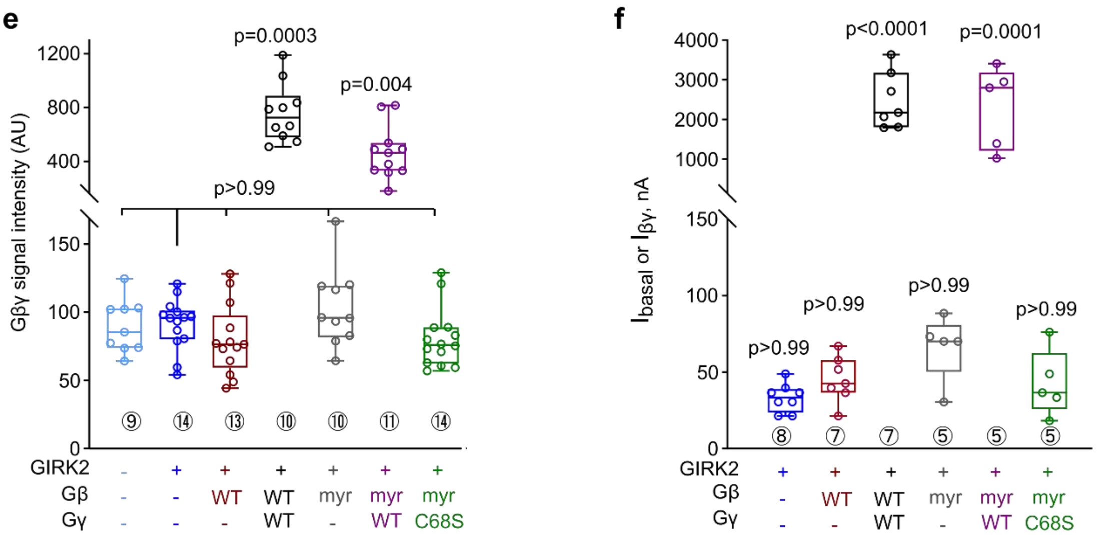

Supplementary Fig. 1. GIRK2 activation by ACh and Gβγ and the requirement for prenylation of Gγ. a, I_{basal}, fold activation by ACh (R_a) and fold activation by Gβγ ($R_{\beta\gamma}$). The table summarizes experiments in which, on the same day, I_{basal} and I_{evoked} were measured in one group of oocytes expressing m2R and GIRK2, and $I_{\beta\gamma}$ in another group of expressing Gβγ, in 24 mM $[K^+]_{out}$ solution. Results were grouped according to $I_{\beta\gamma}$ as indicated in the 2nd column. m2R RNA was 0.5-1 ng to maximize I_{evoked}[2]. Gβ (5 ng RNA) was coexpressed with Gγ or Gγ-YFP (1 or 2-2.5 ng, accordingly). **b**, WB (2 oocytes/lane) shows that YFP-$G\gamma_{C68S}$ protein expression in whole oocytes is comparable with YFP-$G\gamma_{WT}$. The same is true for the coexpressed $G\beta_{WT}$. The $G\beta_1$ (GNB1) antibody marginally recognizes the oocyte's endogenous Gβ (faint lane next to the right marker). **c**, summary of western blots as in **a** of YFG-Gγ (2 ng RNA) and Gβ (5 ng RNA) expressed in whole oocytes (n=4), normalized to control ($G\beta\gamma_{WT}$). Net expressed Gβ was calculated by subtracting the endogenous Gβ signal. Statistics: one-sample two-sided t-test vs. theoretical mean of 1. **d**, examples of GMPs from oocytes injected with the indicated RNAs (YFP-GIRK2 was used), stained with a Gβ antibody. Image sizes are 272×272 µm. RNAs injected were (in ng): YFP-GIRK2, 5; Gβ, 5; Gγ or $G\gamma_{C68S}$, 2, m2R, 1. Only $G\gamma_{WT}$, but not $G\gamma_{C68S}$, ensures the PM attachment of Gβ or myr-Gβ. A weak signal, reflecting the PM-attached endogenous Gβ, is observed in uninjected (naïve) oocytes or after expression of Gβ or myr-Gβ without Gγ. Note that myr-Gβγ is a functional protein that reaches PM and activates GIRK2 when expressed with $G\gamma_{WT}$. **e**, summary of Gβ measurements in GMPs. Statistics: Kruskal-Wallis test followed by Dunn's multiple comparison vs. native oocytes. **f**, summary of GIRK currents measured in 24 mM $[K]_{out}$ in the same experiment as d, e. Statistics: as in c. In **e** and **f**, number of cells tested is shown near data boxes (encircled numbers).

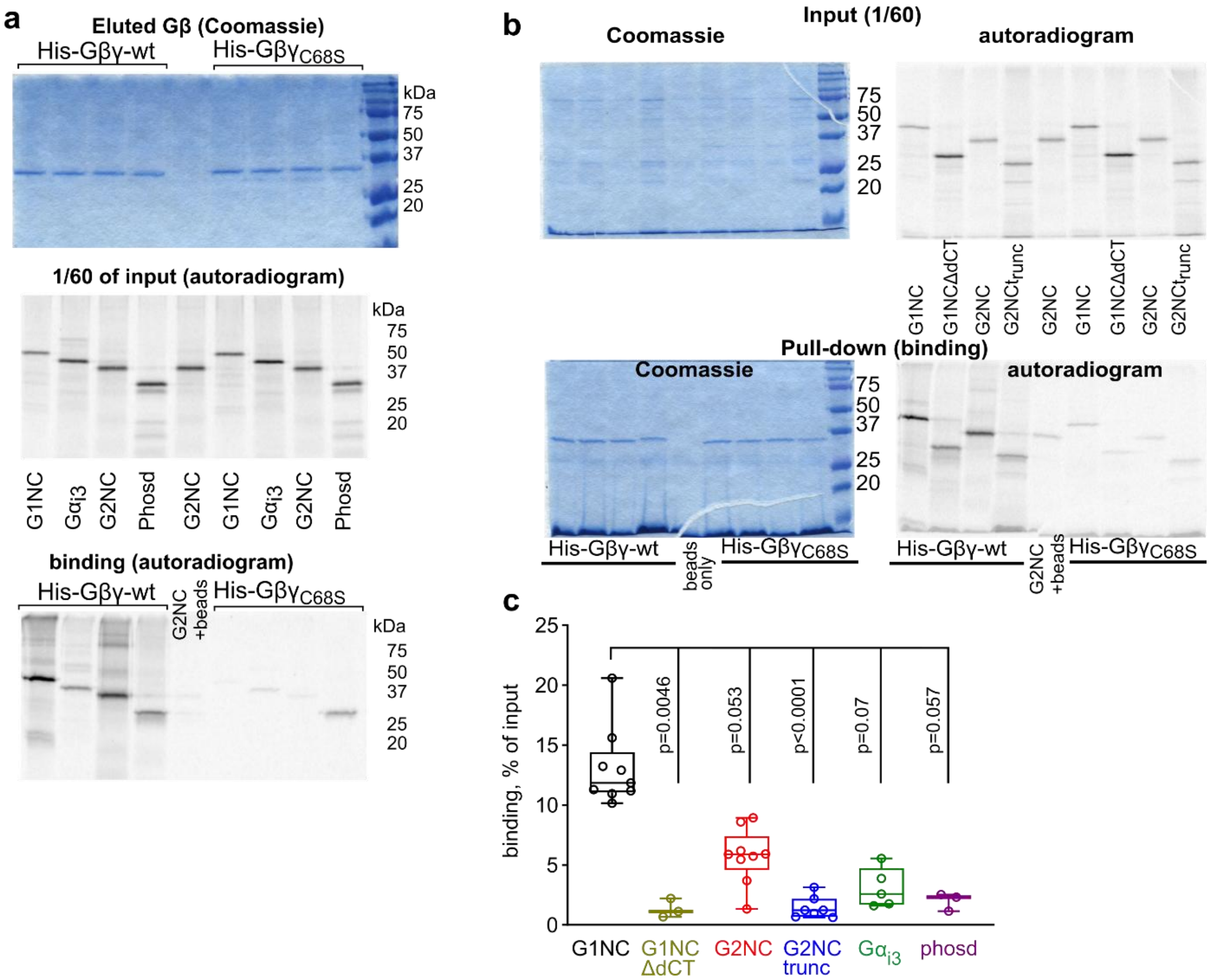

Supplementary Fig. 2. Differences in binding of Gα$_{i3}$-GDP, phosducin and cytosolic segments of GIRK1 and GIRK2 to WT Gβγ and non-prenylated Gβγ. Each one of the gels shown is representative of at least 3 experiments. **a,** the full gel of the experiment shown in Fig. 1c. **b**, a representative experiment comparing binding of *ivt* full-length G1NC and G2NC and their truncated versions, G2NC$_{trunc}$ and G1NC$_{ΔdCT}$, to WT His-Gβγ and non-prenylated His-Gβγ$_{C68S}$. Upper and lower images represent two separate gels. Left images show Coomassie staining of proteins in reaction mix (1/60 of total; "input (1/60)", top) and eluted proteins (binding; bottom). Right images show autoradiograms of the same gels. **c**, comparison of binding of the various interactors to Gβγ$_{WT}$. Shown are the same data as in Fig. 1d but without the binding to Gβγ$_{C68S}$. The difference between G1NC and G1NC$_{ΔdCT}$ was highly significant, p=0.0046, but for G2NC and G2NC$_{trunc}$ it was not significant, p=0.117. Statistics: Kruskal-Wallis test followed by Dunn's multiple comparison test vs. control (G1NC).

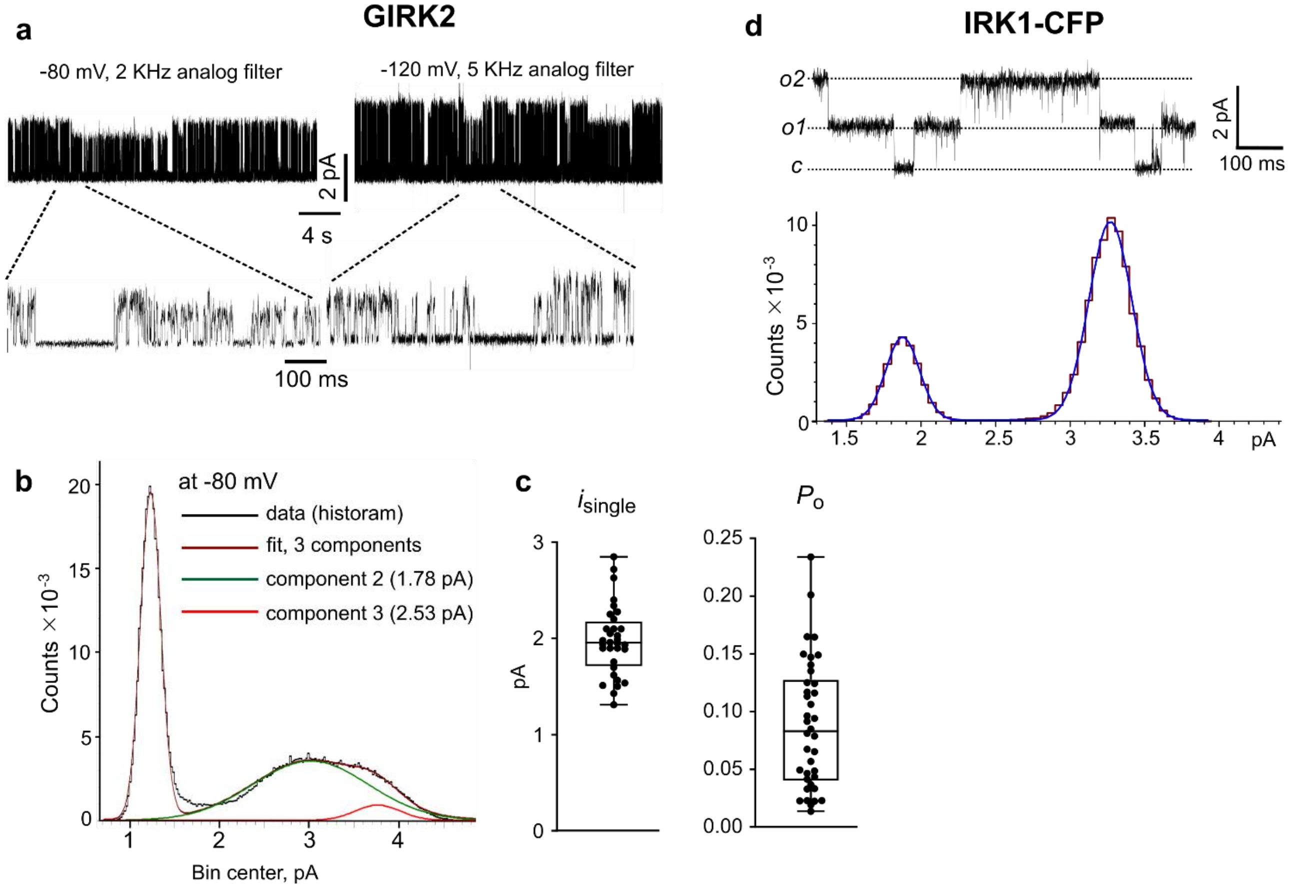

Supplementary Fig. 3. Characterization of single-channel parameters of GIRK2 and IRK1-CFP. a-c, analysis of a cell-attached record from an oocyte injected with 25 ng of the anti-GIRK5 oligonucleotide and the following RNAs (in ng/oocyte): GIRK2, 0.017; m2R, 2; Gβ, 5; Gγ, 1. The patch contained one active channel, as assessed from lack of overlaps during the ~10-minute record. **a,** representative segments of the record at -80 mV and -120 mV acquired at 20 KHz with either 2 KHz or 5 KHz analog filter, as indicated. Inward K^+ currents are shown as upward deflections. **b,** all-points amplitude histogram of the left record from **a** (at -80 mV). The left peak corresponds to the background noise. The parameters of the 3 components of the Gaussian fit were: μ_1=1.23 pA, σ_1=0.11 pA; μ_2=3.01 pA, σ_2=0.64 pA; μ_3=3.76 pA, σ_3=0.25 pA. The two components of the fit corresponding to the two subconductance levels are shown with green and red lines. Two conductance levels (substates) have been observed in a minority of GIRK2 records. The proportion of the two levels varied among patches, but usually either the larger or the smaller one was predominant (see, for example, Fig. 3c,d). In most cells the channel current in all-point amplitude histograms was well fitted with one Gaussian component. **c,** single channel parameters of GIRK2. Box plots: 25-75 percentile range; whiskers – full range; middle line – median. P_o was calculated from 2 to 4 min segments of idealized traces from patches containing 1 to 3 channels. The weighted averaged i_{single} was calculated from all-points histograms. Amplitude analysis was limited to patches with P_o>0.05 to avoid filtering artifacts with very short openings. **d**, section of a record from an oocyte injected with 5 pg IRK1-CFP RNA. Holding potential was -80 mV. Two channels were present; *c* denotes closed channel current level, *o1* – one open channel, *o2* – two open channels' current. *Bottom*, all-points histogram of a section of the record from the same patch, fitted with a two-component Gaussian for determining the single channel amplitude, i_{single}. In this patch, P_o was 0.81 and i_{single} was 1.39 pA. A summary of single channel parameters for all channels used here is presented in Supplementary Table 3.

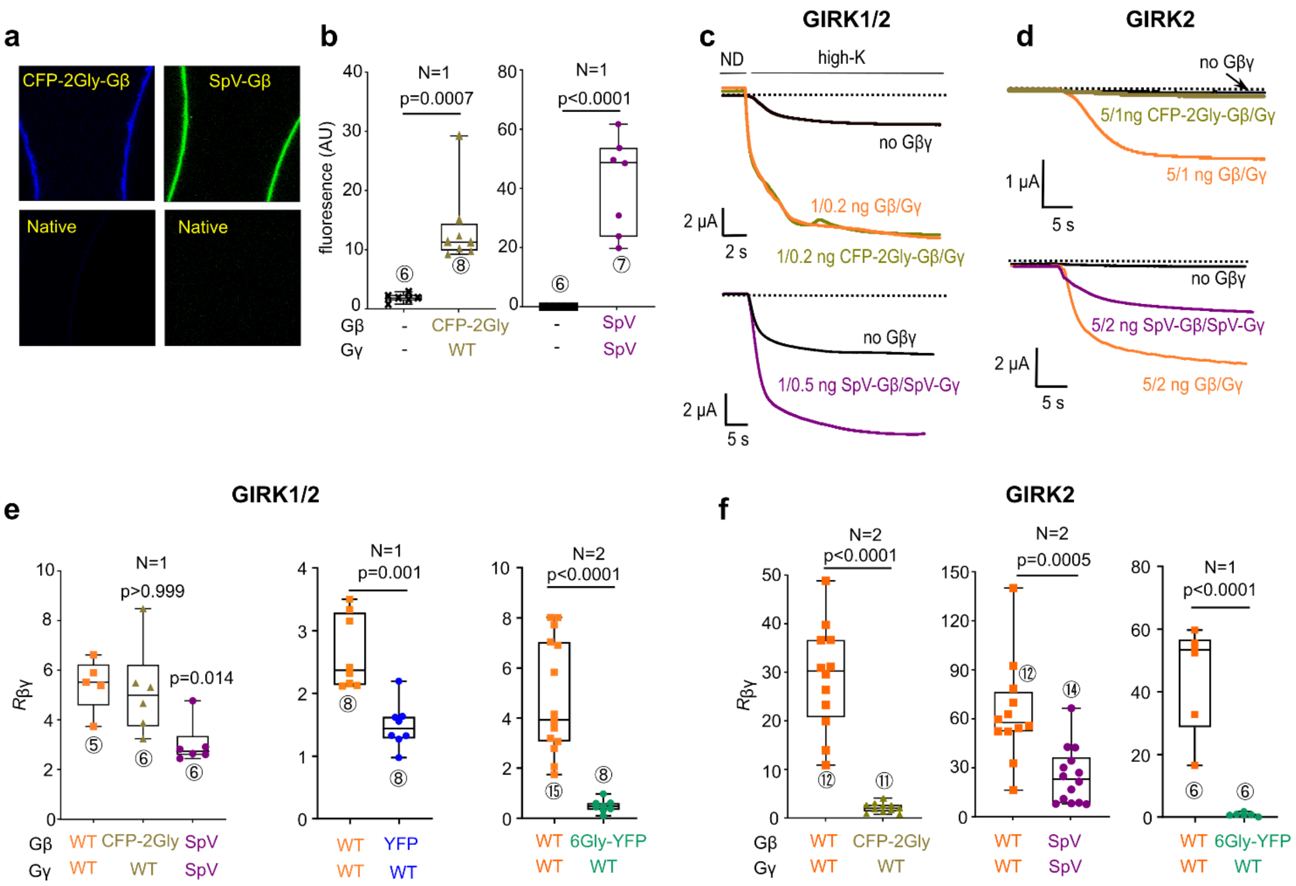

Supplementary Fig. 4. xFP-fused Gβ constructs perform poorly in activating GIRKs. a, examples of confocal images of oocytes injected with GIRK2 (2 ng RNA) and RNAs of CFP-2Gly-Gβ (1 ng) and Gγ (0.4 ng), or SpV-Gβ (1 ng) and SpV-Gγ (0.5 ng). Each image is 272×272 μm. Both SpV-Gβγ and CFP-2Gly-Gβ were expressed in the plasma membrane. The intensity of images from the CFP-2Gly-Gβ experiment (both native and Gβ-expressing oocytes) was enhanced 2-fold using Corel PhotoPaint, for better visibility. **b,** summary of expression levels of CFP-2Gly-Gβ and SpV-Gβγ in whole oocytes, compared to the background fluorescence of native oocytes. AU, arbitrary units. Two-tailed unpaired t-test was used for CFP-2Gly-Gβ and Mann-Whitney test was used for SpV-Gβγ. **c, d**, representative whole-cell currents in oocytes expressing GIRK1/2 (**c**) and GIRK2 (**d**). The amounts of injected RNAs were: GIRK1, 0.05 ng; GIRK2, 0.05 ng as heterotetramer and 2 ng as homotetramer. The amounts of Gβ or xFP-Gβ were: 1 ng when coexpressed with GIRK1/2 and 5 ng with GIRK2. The record started in a low-K^+ external solution (ND96; ND) which was then switched to high-K solution (24 mM $[K^+]_{out}$). **e, f**, summary of fold-Gβγ activation ($R_{\beta\gamma}$) by Gβγ vs. the various xFP-labeled Gβ with WT Gγ (except for SpV Gβγ). Gβ RNA doses were as in **c**, except Gβ-6Gly-YFP, which was 5 ng RNA/oocyte in all cases. Of all constructs, only CFP-2Gly-Gβ activated GIRK1/2 similarly to Gβγ (**e**), but it did not activate GIRK2 (**f**). SpV-Gβγ activated both GIRK2 and GIRK1/2 but significantly less than $G\beta\gamma_{WT}$. With Gβ-6Gly-YFP (YFP fused to Gβ's C terminus via a 6-glycine linker), no activation at all was seen with GIRK2 (**f**, right panel), and GIRK1/2 was even reduced relative to its I_{basal} (**e**, right panel; $R_{\beta\gamma}$=0.49±0.09). Statistical analysis included the Shapiro-Wilk normality test, and the following analysis was done using the appropriate tests as detailed in Methods: Kruskal-Wallis test followed by Dunn's test (vs. WT) in **e**, and two-tailed unpaired t-test or Mann-Whitney test in the rest. Number of cells (encircled) is shown near the columns, and number of experiments is shown as N. In all cases, in box plots the box is 25-75 percentile range; whiskers – full range; middle line – median.

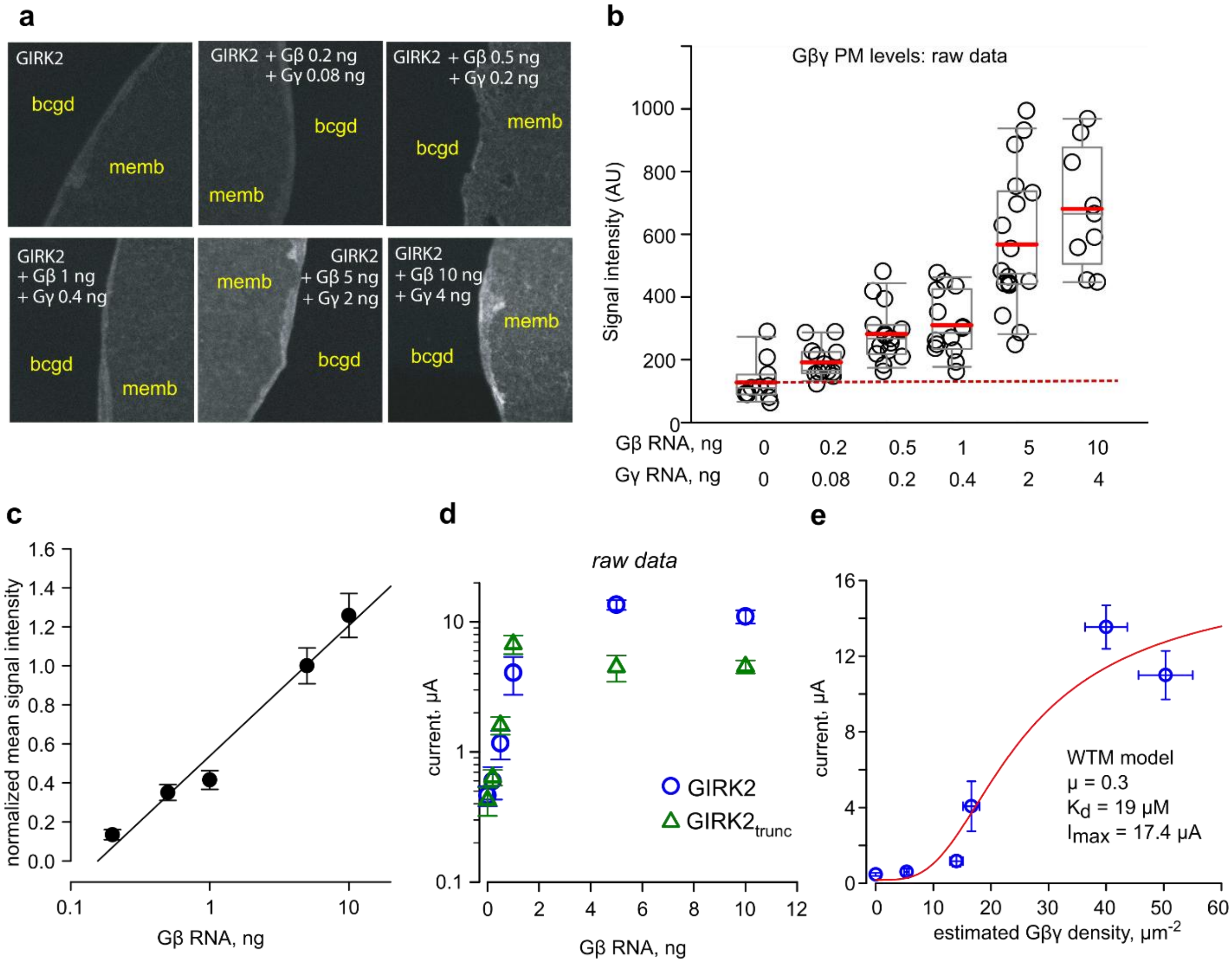

Supplementary Fig. 5. Dose-dependent Gβγ activation of GIRK2 and GIRK2$_{trunc}$. Whole-cell currents of the two constructs were compared in one experiment. **a,** representative images of giant plasma membrane patches (GMP) stained with Gβ antibody (Santa Cruz, SC-378). Each image is 272×272 μm. GIRK2 channel (2 ng RNA/oocyte) was present in all groups, and Gβ and Gγ RNAs were coinjected in the indicated amounts. bckg, background; memb, membrane. **b**, summary of measurements of Gβγ in GMPs. AU, arbitrary units. Nine to 18 GMPs were measured with each Gβγ dose. Boxes show 25-75 percentiles and whiskers 5-95 percentiles. Red lines within the boxes show the mean and the grey lines the median values. **c**, relation between the amount of injected Gβ RNA and Gβ protein measurement in the GMPs. Circles show mean±SEM from the same measurements shown in b. In this experiment, the surface levels of expressed Gβγ were empirically found to be linearly related to log[Gβ RNA dose]. Data are shown as mean±SEM, with linear regression line. Net values of expressed Gβγ, after subtraction of the average background signal measured in native oocytes, were normalized to the signal seen with 5 ng Gβ RNA. **d**, whole-cell GIRK2 and GIRK2$_{trunc}$ (2 ng RNA/oocyte) currents, shown as mean±SEM, measured in 96 mM K^+ solution. GIRK2$_{trunc}$ lacks the first 51 amino acid residues (a.a.) of the cytosolic N-terminus and the last 24 a.a. of the CT. **e,** dose-dependent activation of GIRK2 by Gβγ. The density of expressed Gβγ in the PM obtained with 5 ng Gβ RNA was assumed to be 40 molecules/μm^2 (as in Fig. 3 and close to the average 35 molecules/μm^2 from qWB data, Supplementary Table 4), and Gβγ PM densities for other RNA doses were calculated based on the regression line from Fig. 2c. The circles show mean estimated Gβγ density (±SEM) on the X axis (n=8-18) and mean±SEM current on the Y-axis (n=7-13). The parameters of WTM model fit with fixed μ=0.3 (red line) are shown in the inset.

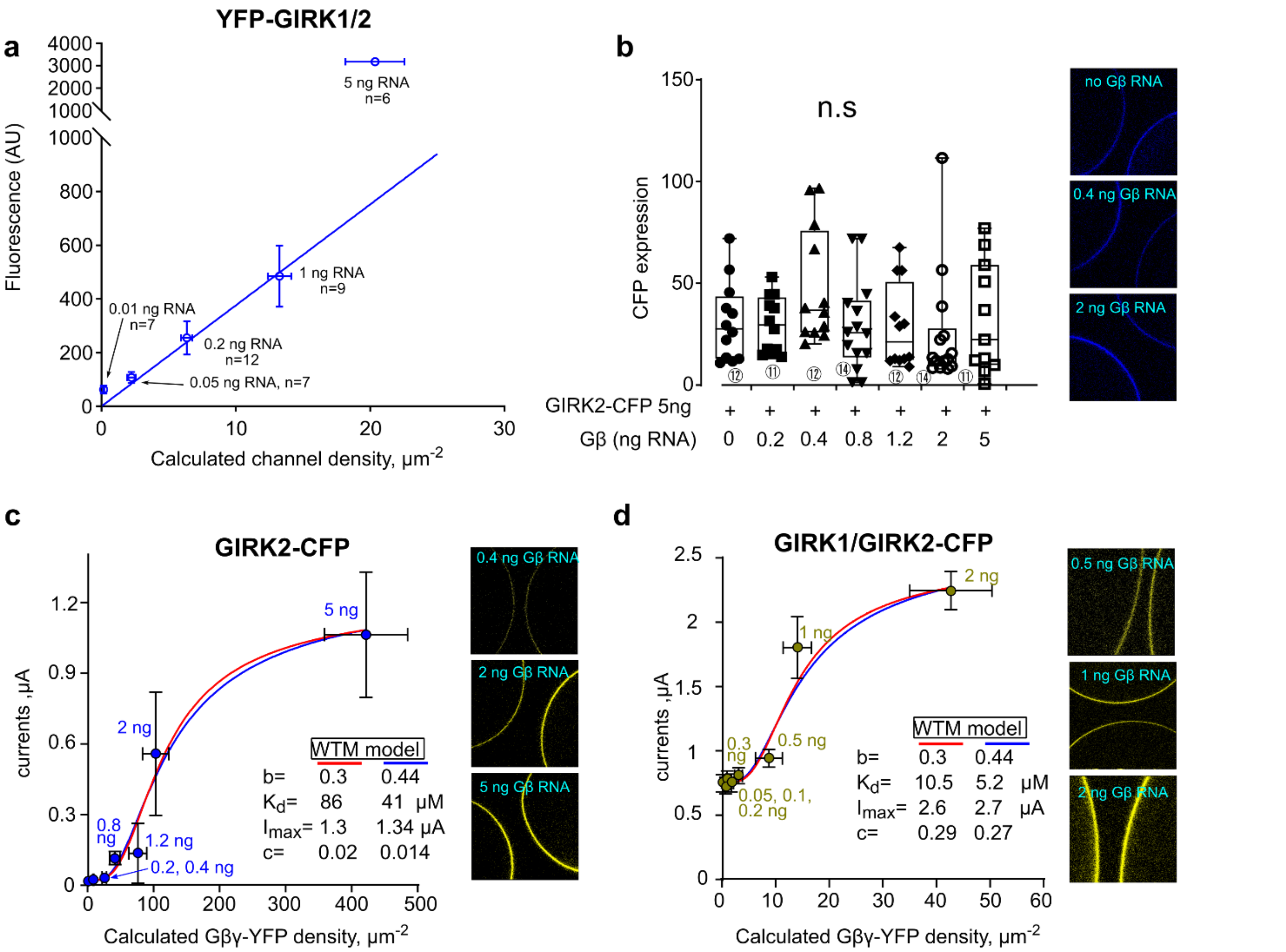

Supplementary Fig. 6. Testing linear range of CE calibration (a) and dose-dependent activation of GIRK2-CFP and GIRK1/2-CFP by coexpressed $G\beta\cdot_{YFP}G\gamma$ (b-d). a, testing linearity of surface fluorescence of YFP vs. surface density of YFP-GIRK1/2 in a wide range of channel subunits' RNA (0.01-5 ng/oocyte of each subunit, shown near each point) coexpressed with Gβγ (5:1 ng RNA). YFP fluorescence was measured in 10 oocytes in each group; for current measurements, n is shown near the points. Data are shown as mean±SEM. The linear regression line was drawn via zero and all data points except 5 ng RNA (we never used more than 1 ng in GIRK1/2 experiments). Deviations from linearity were observed in all 3 experiments where 5 ng GIRK1/2 RNAs were tested. **b-d**, data are from one experiment. Calibration was done with YFP-GIRK1/2 coexpressed with Gβγ (5:1 ng RNA; not shown). **b**, GIRK2-CFP (5 ng RNA/oocyte) was expressed with $G\beta\cdot_{YFP}G\gamma$ (2:1 ratio Gβ to YFP-Gγ RNA), and its expression was measured from confocal images of intact oocytes (right panel; image size 544×544 μM). Coexpression of $G\beta\cdot_{YFP}G\gamma$ did not significantly affect the expression of GIRK2-CFP in the PM. Boxes show 25-75 percentiles, whiskers the full data range, lines within the boxes show the median. Numbers of cells (encircled) are shown below boxes. Statistics: Kruskal-Wallis ANOVA followed by Dunn's multiple comparison test. **c**, dose-dependent activation of GIRK2-CFP by $G\beta\cdot_{YFP}G\gamma$. The expression of Gγ-YFP was measured in 11-14 intact oocytes for each group, as in **b** (exemplary images are in the right panel; image size 550×550 μM). GIRK currents were measured in 8-10 oocytes. WTM fits with μ=0.3 (red line) and μ=0.44 (blue line) are shown. **d,** dose-dependent activation of GIRK1/GIRK2-CFP (50 pg RNA/oocyte of each subunit) by $G\beta\cdot_{YFP}G\gamma$. $G\beta\cdot_{YFP}G\gamma$ expression in the PM was measured from 9-12 intact oocytes for each $G\beta\cdot_{YFP}G\gamma$ dose. Representative images are shown on the right (image size 272×272 μM). GIRK currents were measured in 5-18 oocytes. WTM fits with μ=0.3 (red line) and μ=0.44 (blue line) are shown. This result was not included in the summary of Fig. 4h and Supplementary Table 6, because GIRK2-CFP showed lower sensitivity to Gβγ than WT GIRK2, as judged both by the small magnitude of $I_{\beta\gamma}$ and the high K_d.

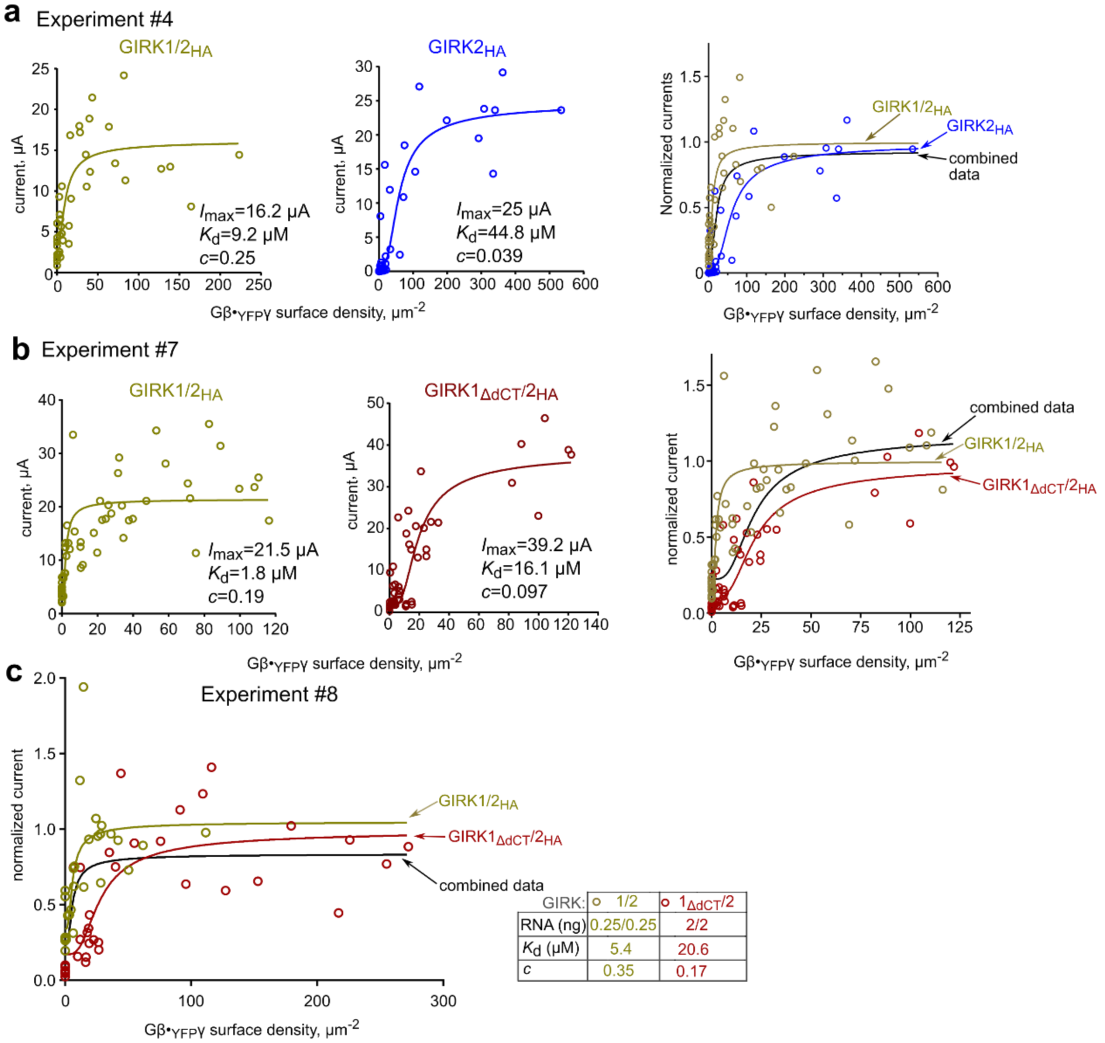

Supplementary Fig. 7. GIRK2 and GIRK1$_{\Delta dCT}$ show lower apparent affinity to Gβγ than GIRK1/2: raw data in individual oocytes and WTM fits. Data (experiments 5 and 7 as in Fig. 4g) were fitted with the WTM model with µ=0.3; fit parameters are in the panels. **a,** activation of GIRK1/2$_{HA}$ (left; 0.25 ng RNA of each subunit) and GIRK2$_{HA}$ (middle; 5 ng RNA/oocyte) by Gβ·$_{YFP}$Gγ (0.2-10 ng Gβ, 5:1 Gβ:Gγ RNA). Right panel: WTM fits of currents normalized to maximal $I_{\beta\gamma}$ (I_{max}, Supplementary Table 6) and fitted as in Fig. 4 but, in this case, we also included a comparison with the combined data (experimental points for both channel compositions) corresponding to null hypothesis (no difference between the dose-response curves). The analysis was done according to GraphPad guide: https://www.graphpad.com/guides/prism/latest/curve-fitting/reg_comparing_fits_with_anova.htm. Briefly, the data for two channel compositions were fitted separately and together (combined data) to Eq. 5. The K_d, n and the SEM parameter of goodness of fit from the three fits were compared using one-way ANOVA and the p value was extracted from F-test and AICc test reports. The differences between the three sets were significant, $F(2,162)=146460$, $p<0.0001$ by F test; $P<0.01$ by AIC test). **b**, GIRK1/2$_{HA}$ (left;, 0.25 ng RNA/oocyte) and GIRK1$_{\Delta dCT}$/2-HA (middle; 2 ng RNA/oocyte) were activated by coexpression of Gβ (0.1-5 ng RNA/oocyte) and YFP-Gγ in 2:1 RNA ratio. Right panel: WTM fits of normalized currents (as in **a**) fitted to the WTM model (µ = 0.3) for GIRK2$_{HA}$, GIRK1/2$_{HA}$, and the combined data. The differences between fits were significant: $F(2,206)=28358$, $P<0.0001$; $P<0.01$ by AIC test. **c**, dose-dependent activation of GIRK1/2$_{HA}$ and GIRK2$_{HA}$ by Gβ·$_{YFP}$Gγ (experiment #8). Analysis and presentation of data are as the right panels in a and b. The differences between the fits were significant, $F(2,116)=24243$, $P<0.0001$. In a pairwise comparison between GIRK1/2$_{HA}$ and GIRK2$_{HA}$, the difference in K_d was significant: $F(1, 58)=12.15$, $p=0.0009$.

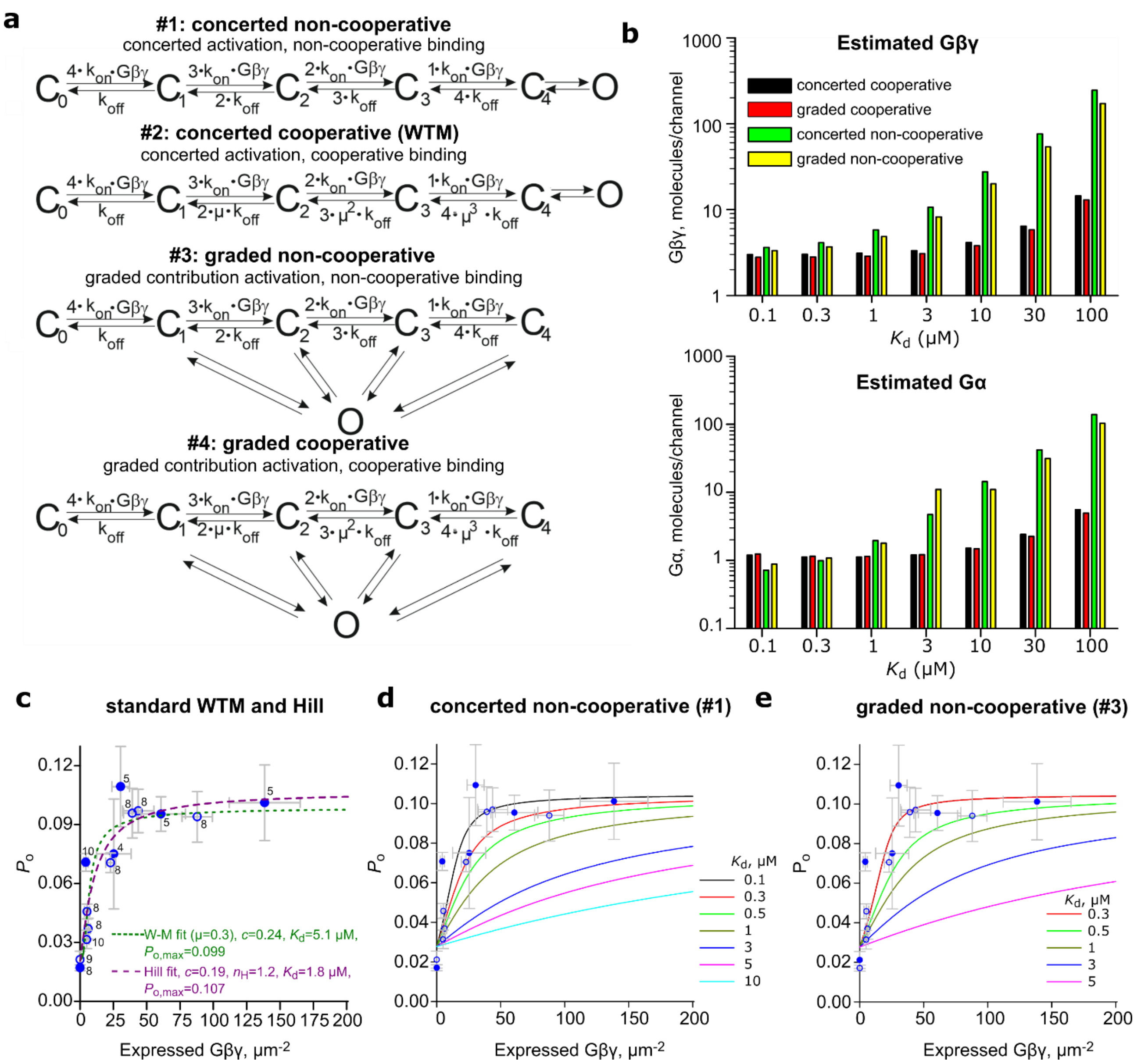

Supplementary Fig. 8. Modeling basal and Gβγ-evoked GIRK1/2 activity. a, the four kinetic models for Gβγ activation of GIRK1/2. Up to four Gβγ bind sequentially to the activation sites. In concerted models, the channel opens only when all four sites are Gβγ-occupied. In graded contribution models, occupation of the first site leads to opening, and binding to each additional site increases the P_o in a more-than-additive manner[1]. In cooperative models, each subsequent Gβγ binds with a higher affinity than the previous one. In the non-cooperative models, the affinity of Gβγ to each binding site is the same. **b**, amounts of available basal endogenous Gα and Gβγ that determine I_{basal} and I_{evoked}, per channel, were calculated with each of the kinetic models for a range of K_d (see Supplementary Methods). **c**, data from two experiments with GIRK1/2$_{HA}$ (Supplementary Table 6), #4 (closed symbols) and #7 (open symbols; experiment of Fig. 4d), were pulled and fitted to the standard WTM and Hill models. Each point shows mean±SEM of P_o (Y-axis) vs. Gβ$\cdot_{YFP}$γ surface density (X-axis). Numbers of cells are shown near the data points. The whole-cell current at each Gβγ dose was expressed as fraction of the maximal current obtained in WTM fit (24 and 13.4 μA in experiments #4 and #7) and transformed into P_o assuming $P_{o,max}$=0.105 (Supplementary Table 3). **d**, **e**, simulated Gβγ dose-response curves for the concerted and graded non-cooperative models, with a range of K_d values. The basal P_o of ~0.3 is not a fitted value but emerges from basal levels of Gβγ and Gα (as shown in **b**). The data points are the same as in **c.** The simulations best align with data with K_d between 0.1-0.5 μM.

a

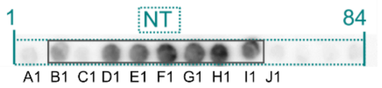


```
B1 RKFGDDYQVVTTSSSGSGLQPQGPG
C1 DYQVVTTSSSGSGLQPQGPGQGPQQ
D1 TTSSSGSGLQPQGPGQGPQQQLVPK
E1 GSGLQPQGPGQGPQQQLVPKKKRQR
F1 PQGPGQGPQQQLVPKKKRQRFVDKN
G1 QGPQQQLVPKKKRQRFVDKNGRCNV
H1 QLVPKKKRQRFVDKNGRCNVQHGNL
I1 KKRQRFVDKNGRCNVQHGNLGSETS
```

b

```
hGIRK2    1     makltesm tnvlegdsmd qdvespvaih qpklpkqard dlprhisrdr tkrkiqryvr kdgkcnvhhg nvretyrylt  78
mGIRK2A   1   mtmakltesm tnvlegdsmd qdvespvaih qpklpkqard dlprhisrdr tkrkiqryvr kdgkcnvhhg nvretyrylt  80

hGIRK2    79  difttlvdlk wrfnllifvm vytvtwlffg miwwliayir gdmdhiedps wtpcvtnlng fvsaflfsie tettigygyr  158
mGIRK2A   81  difttlvdlk wrfnllifvm vytvtwlffg miwwliayir gdmdhiedps wtpcvtnlng fvsaflfsie tettigygyr  160

hGIRK2    159 vitdkcpegi illliqsvlg sivnafmvgc mfvkisqpkk raetlvfsth avismrdgkl clmfrvgdlr nshiveasir  238
mGIRK2A   161 vitdkcpegi illliqsvlg sivnafmvgc mfvkisqpkk raetlvfsth avismrdgkl clmfrvgdlr nshiveasir  240

hGIRK2    239 aklikskqts egefiplnqt dinvgyytgd drlflvspli isheinqqsp fweiskaqlp keeleivvil egmveatgmt  318
mGIRK2A   241 aklikskqts egefiplnqt dinvgyytgd drlflvspli isheinqqsp fweiskaqlp keeleivvil egmveatgmt  320

hGIRK2    319 cqarssyits eilwgyrftp vltledgfye vdynsfhety etstpslsak elaelasrae lplswsvssk lnqhaelete  398
mGIRK2A   321 cqarssyits eilwgyrftp vltledgfye vdynsfhety etstpslsak elaelanrae lplswsvssk lnqhaelete  400

hGIRK2    399 eeeknleeqt erngdvanle neskv 423
mGIRK2A   401 eeeknpeelt erng             414
```

Supplementary Fig. 9. Illustration of analysis of peptide array data and comparison of hGIRK2 with mGIRK2A. a, the method used to designate the approximate boundaries of Gβγ-binding regions from peptide array scans, exemplified with the GIRK1 NT Gβγ-binding region (highlighted in yellow). The boundaries were arbitrarily defined before the last 10 a.a. of the first 25-mer Gβγ-labeled peptide, and after the first 10 a.a. of the last Gβγ-labeled peptide. In cases when the starting or the last peptide of the array bound Gβγ, 5 N-terminal a.a. were not counted as part of the binding site. **b**, alignment of a.a. sequences of mouse GIRK2A (mGIRK2A) used in most experiments in this report, and hGIRK2 used for the peptide arrays. There are 2 a.a. differences (highlighted in yellow). In addition, hGIRK2 is 2 a.a. shorter in its N terminus and contains 11 a.a. in its dCT not present in mGIRK2A. The last a.a. of mGIRK2A (G414) corresponds to G412 of hGIRK2.

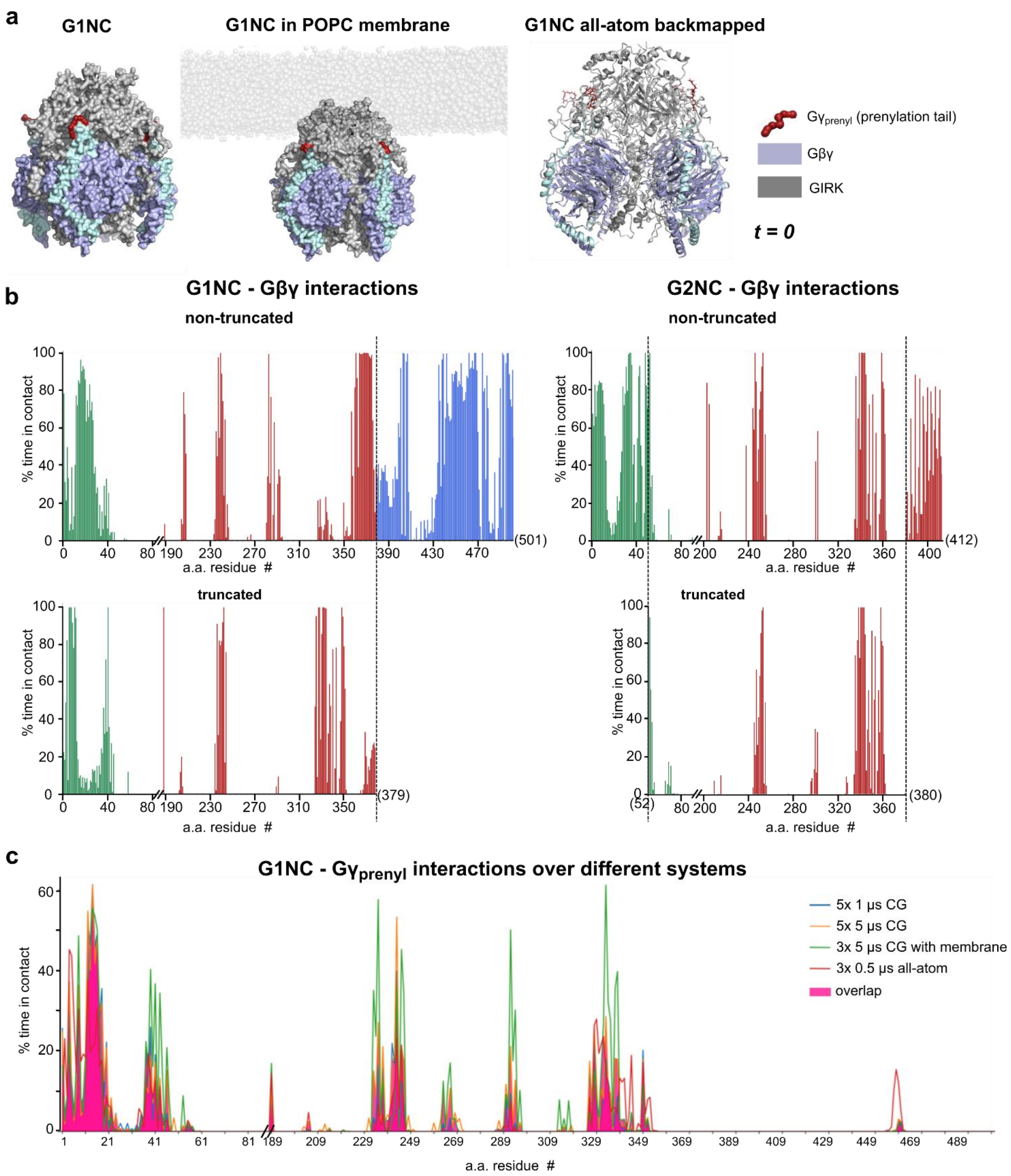

Supplementary Fig. 10. MD simulations of binding of prenylated Gβγ to full-length and truncated G1NC and G2NC, and of $G\gamma_{prenyl}$ to Gβ. a, the experimental systems of Gβγ-bound G1NC used in coarse-grained (left and center) and all-atom (right) simulations, at the beginning of the simulation (t=0). See Supplementary Table 11 for details of the systems. **b**, histograms showing % of time spent by each a.a. within full-length (non-truncated) and truncated G1NC and G2NC in contact with Gβγ across all 1-μs runs. Highly similar data were obtained in 5 μs runs (see Supplementary MD Figures Collection). **c**, summary of interactions of $G\gamma_{prenyl}$ with G1NC in the different simulation systems.

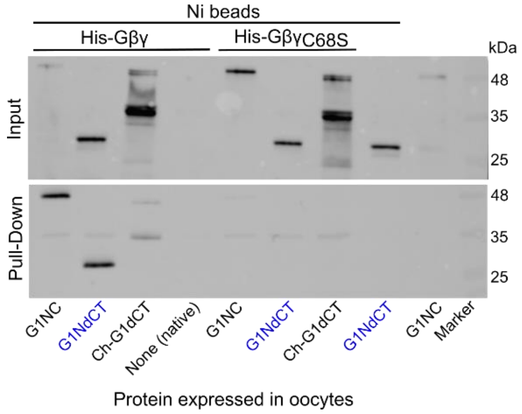

Supplementary Fig. 11. Gγ prenylation is essential for strong binding of G1NdCT. One experiment, representative of two. The indicated proteins were expressed in *Xenopus* oocyte. Whole-cell lysates (2 oocytes/sample) were incubated with purified His-Gβγ or His-Gβγ_{C68S} and pull down was performed on Ni-NTA beads in the same way as for reticulocyte lysates (see Methods and Fig. 1). Proteins were detected by WB with the Alomone anti-GIRK1 antibody #APC-005 directed against G1-dCT (residues 437-501) https://www.alomone.com/p/anti-kir3-1-girk1/APC-005?srsltid=AfmBOoqGejtRcAbA032YgyGe_eqlYWogDoN1ZVGYcBneKrkQ6AqevvAB#specification. Note that mCherry-fused G1dCT (Ch-G1dCT) binds His-Gβγ only weakly, despite higher amounts of this protein synthesized by the oocytes (Input). His-Gβγ_{C68S} binds all proteins much weaker than His-Gβγ_{WT}.

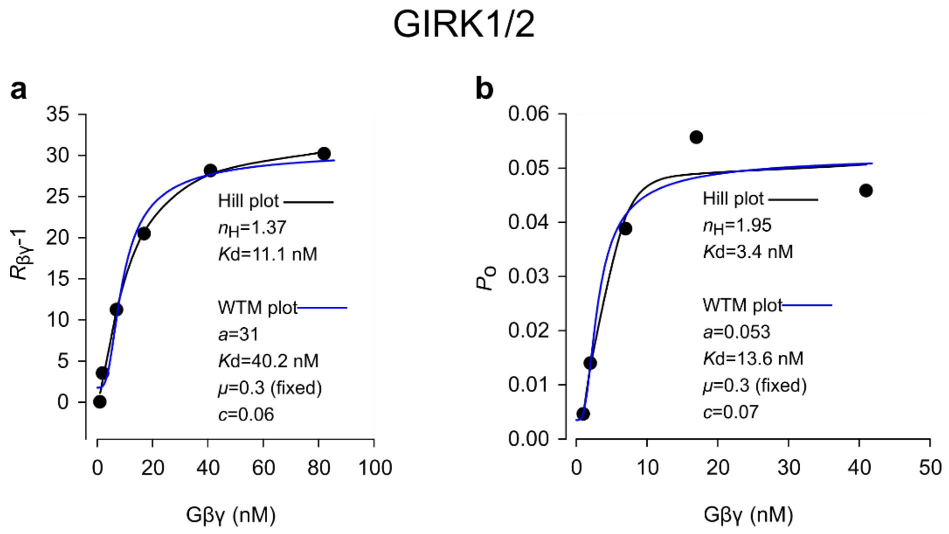

Supplementary Fig. 12. Dose-dependent activation of GIRK1/2 by Gβγ added to the bathing solution in excised patch experiments. Original data reported in Peleg et al., 2002[23] were fitted to Hill and WTM models. **a**, results from multichannel patches (>3 channels/ patch; n=4-8 patches for each point). $R_{\beta\gamma}$ is calculated as (NP_o at the peak of activation by Gβγ)/(NP_o during the last minute before addition of Gβγ). Channel activity is presented as $R_{\beta\gamma}$-1. **b**, results from patches with 1-3 channels where the exact P_o could be calculated (n=3-8 except the lowest does of Gβγ, where n=1). For additional details, see Fig. 7 in Peleg et al. 2002[23]. Note that basal activity of GIRK1 these patches was 3-7% of maximal P_o, which is lower than in our usual whole-cell records. There are two reasons for that: first, activation by Gβγ is stronger for low expression levels of GIRK1/2[1, 23] (to achieve low channel density for single-channel recordings, we injected 5-20 pg RNA vs. 50-250 pg in standard whole-cell experiments, e.g. Fig. 3, Supplementary Fig. 3). Second, Gβγ was applied 3 min after excision when basal activity already decays by ~50% compared to cell-attached mode (Fig. 5). Comparing WTM fits of GIRK1/2 activation by coexpressed Gβγ in whole oocytes (K_d=5.5 μM, Fig. 4f) and by purified Gβγ in excised oocyte's patches[23] (K_d=13-40 nM) suggests a partition coefficient between 140 and 425.

Supplementary Tables

Supplementary Table 1. Dissociation constants of Gβγ effectors, including GIRKs.

Gβγ-binding protein	K_d	Method and Gβγ used	Ref.
$G\alpha^{GDP}$ (various Gα proteins)	0.2-27 nM	Fluorescent flow cytometry with bovine brain Gβγ in detergent solutions	[24]
PH-PLCγ1<br>Ras-GRF<br>SOS-PH	318 nM<br>108 nM<br>208 nM	SPR; **prenylated** $G\beta_1\gamma_1$	[25]
KCTD12 (H1 domain)	185 nM	Isothermal titration calorimetry; **non-prenylated** $G\beta_1\gamma_2$	[26]
D2 (in I-II linker of $Ca_V2.1$ α1 subunit)<br>AID, as above	24 nM<br><br>63 nM	Pull-down of *ivt* $G\beta_1\gamma_2$ (presumably prenylated) by GST-fused segments of I-II linker on glutathion affinity resin	[27]
phosducin	42 nM	SPR; **prenylated** $G\beta_1\gamma_1$	[28]
GRK2 (βARK1)	25 nM	Purified **prenylated** $G\beta_1\gamma_2$ added to GRK2 affinity beads (not in lipid phase)	[29]
PLCβ2	3.2 µM	lipid bilayers, binding curve from FRET, **prenylated** Gβγ concentration in membrane was estimated directly	[30]
cardiac I_{KACh}<br>GIRK1<br>GIRK4	55 nM<br>125 nM<br>50 nM	Immunoprecipitated GIRKs bound to Gβγ on the surface of immunobeads; competition with radiolabeled **prenylated** Gβγ added in detergent solution.	[31]
GIRK4, short C-terminal peptide (209-225)	60 nM	competition of synthetic peptides with atrial GIRK1/4 for binding of **prenylated** $G\beta_1\gamma_2$ in detergent solution	[32]
GIRK4 C-terminus	≤790 nM	SPR; **prenylated** $G\beta_1\gamma_2$	[33]
GIRK1 cytosolic domain, N- and C-terminally truncated	250 µM	NMR in solution; **non-prenylated** $G\beta_1\gamma_2$	[34]
Purified $GIRK2_{trunc}$	1.9 mM (~300 µM in high Na^+)	lipid bilayers, dose-response of channel's activity, **non-prenylate**d $G\beta_1\gamma_2$	[35]

Abbreviations: AID, α-subunit binding domain; βARK1, β-adrenergic receptor kinase 1; GRK2, G protein-dependent receptor kinase 2; NMR, nuclear magnetic resonance; PH, plekstrin homology domain; PLC, phospholipase C; Ras-GRF, Ras-specific guanine nucleotide exchange factor; SOS, son-of-sevenless protein; SPR, surface plasmon resonance.

Supplementary Table 2. The values of K_d calculated for sequential cooperative Gβγ binding to GIRK2.

K_{d1}, K_{d2}, K_{d3} and K_{d4} are K_d values, in µM, for the binding of the 1st, 2^{d}, 3^{d} and 4th Gβγ, respectively. The numbers in yellow rectangle correspond to estimates of Wang et al.[35] with 0 to 4 bound Na^+ ions per GIRK2 channel. The values of K_{d1} in the following rows have been chosen arbitrarily for illustration purposes and are calculated for the case of constant Na^+ concentration with cooperativity factor for Gβγ sequential binding (μ) fixed at 0.3. For calculation of Na^+-dependent changes the cross-cooperativity coefficient between Na^+ and Gβγ was 0.63[35]. Calculations were done according to the WTM model (Eqn. 5, Methods).

Na ions/ channel	K_{d1}	K_{d2}	K_{d3}	K_{d4} (µM)
0	1900	570	171	51
1	1197	359	108	32
2	754	226	68	20
3	475	143	43	13
4	299	90	27	8
	30	9	2.7	0.8
	20	6	1.8	0.54
	10	3	0.9	0.27
	5	1.5	0.45	0.135
	3	0.9	0.27	0.08
	1	0.3	0.09	0.027
	0.5	0.15	0.045	0.0135
	0.2	0.06	0.018	0.0054
	0.05	0.015	0.0045	0.00135

Supplementary Table 3. Single channel parameters of the various channels

	P_o			i_{single} (pA)		
Channel	mean	SEM	n	mean	SEM	n
GIRK2	0.088	0.009	38	1.98	0.065	32
IRK1-xFP	0.81	0.028	5	1.38	0.03	7
$GIRK1/2_{HA}$	0.103	0.022	4	2.64	0.081	5
YFP-GIRK1/2 + Gβγ (5 ng RNA)	0.105[1]			2.8[1]		

Supplementary Table 3. Single channel parameters of the various channels involved in the calculation of channel surface density, calibration of YFP in the PM from whole-cell currents of various channels, and in simulations. We used known P_o and i_{single} for YFP-GIRK1/2 and determined P_o and i_{single} experimentally under identical conditions for xFP-IRK1, GIRK2 and $GIRK1/2_{HA}$. The values of P_o and i_{single} are from cell-attached patches, at V_m=-80 mV, in 146 $[K^+]_{out}$. For GIRK2, average i_{single} was derived from patches with P_o>0.05, to avoid filtering effect with short openings.

Supplementary Table 4. Gβ surface density.

What has been measured	mean	SEM	N
Endogenous Gβγ in uninjected oocytes– this study	30	11	4
Endogenous Gβγ in uninjected oocytes – previous study*	24	4	4
Endogenous Gβγ in uninjected oocytes (all data combined)	28	5	8
Endogenous Gβγ in oocytes expressing GIRK2	34	7	4
Expressed Gβγ in oocytes injected with RNAs of GIRK2+**Gβγ****	35	13	3
Expressed Gβγ in oocytes injected with RNAs of GIRK2+ $G\beta\cdot_{YFP}G\gamma$ **	35	15	3
Expressed Gβγ in oocytes injected with RNAs of GIRK2+ **Gβγ or** $G\beta\cdot_{YFP}G\gamma$ combined**	35	9	6
Expressed YFP-Gβ in oocytes injected with RNA of YFP-Gβ and WT Gγ*	28	6	4

Supplementary Table 4. Gβ surface density, in μm^{-2}, in naïve oocytes, and surface density of expressed Gβ in oocytes injected with RNAs of Gβ or YFP-Gβ (5 ng) and Gγ (1-2ng) or YFP-Gγ (2-2.5 ng). All surface density measurements were done using the quantitative Western blot (qWB) method.

* Yakubovich et al., 2015[1]

** net expression: Gβγ measured in oocytes expressing GIRK2 alone was subtracted from total Gβ reading.

N is the number of experiments.

Supplementary Table 5. n, N from experiments of Figs. 2 and 4.

Fig. 2h,i	Gβγ coexpressed with:			
	GIRK2: n=		GIRK1/2: n=	
Gβγ RNA (ng)	GMP	Whole oocyte	GMP	Whole oocyte
0.2	25	36	8	22
0.5	27	36	19	32
1	42	36	24	33
2	13	37	5	33
5	47	32	24	32
Number of experiments	N=2<br>N=3	N=3	N=1<br>N=2	N=3
Fig. 4a-c	Gβγ coexpressed with:			
	GIRK2: n=		GIRK1/2: n=	
Gβγ RNA (ng)	surface density	whole-cell current	surface density	whole-cell current
0	-	7	-	8
0.2	10	10	12	10
0.5	11	9	6	4
1	10	9	8	5
2	9	5	8	5
5	8	3	8	5
10	10	5	-	-
Number of experiments	N=1 (all data from one experiment)			

For Fig. 4a-c: In this analysis, we included oocytes measured for both YFP-Gγ fluorescence and $I_{βγ}$, as well as those assessed only for YFP-Gγ expression. 5 ng Gβ was present in all experiments. In the upper table the numbers highlighted in yellow and in cyan correspond to two different sets of experiments.

Supplementary Table 6. The complete fitting results to the WTM model

GIRK2					WTM model, μ=0.3								channel density, μm^{-2}
					Individual cells			Groups			Summary		
**Exp. #**	**method**	**calibration**	**GIRK2**	**See Fig.**	***c***	***K*d (μM)**	***I*max (μA)**	***c***	***K*d (μM)**	***I*max (μA)**	***K*d (μM)**	***c***	
1	c.a. patch	YFP-GIRK1/2	wt	3	0.037	17.1	Po=0.19				17.3	0.037	(0.91)
2	whole cell	YFP-GIRK1/2	HA					0.044	58.5	8.6	58.4	0.044	12.1
3	whole cell	IRK1-YFP	HA		0	14.7	15.4				14.7	0	21.6
4	whole cell	IRK1-YFP	HA	4a-c	0.039	44.8	25	0.052	45.0	24	44.8	0.039	35.1
5	whole cell	Gβγ from GMPs	wt	Supp. 5				0.010	18.4	17.4	18.4	0.01	12.7
6	whole cell	IRK1-YFP	wt		0.050	34.3	5.63	0.000	23.8	5.1	34.3	0	4.1
				**mean**	**0.032**	**27.7**	**15.3**	**0.027**	**36.4**	**13.8**	**31.3**	**0.022**	**17.1**
				**n**	**4**	**4**	**3**	**4**	**4**	**4**	**6**	**6**	**5.0**
				**SEM**	**0.009**	**6.2**	**4.56**	**0.011**	**8.1**	**3.7**	**6.6**	**0.008**	**5.3**

GIRK1/2					WTM model, μ=0.3							
4	whole cell	IRK1-YFP	HA	4a-c	0.250	9.2	16.2	0.24	7.0	14.8	9.2	0.25
7	whole cell	IRK1-YFP	HA	4d	0.190	1.8	21.5	0.17	5.1	24	1.81	0.19
8	whole cell	IRK1-YFP	wt		0.350	5.4	12.8	0.31	5.0	13.4	5.4	0.35
				**mean**	**0.263**	**5.5**	**16.8**	**0.24**	**5.7**	**17.4**	**5.5**	**0.263**
				**n**	**3**	**3**	**3**	**3**	**3**	**3**	**3**	**3**
				**SEM**	**0.038**	**1.7**	**2.06**	**0.033**	**0.5**	**2.7**	**1.7**	**0.038**

GIRK1$_{\Delta dCT}$/GIRK2					WTM model, μ=0.3							
7	whole cell	IRK1-YFP	HA	4d	0.097	16.1	39.2	0.06	14.1	38.9	16.125	0.097
8	whole cell	IRK1-YFP	wt		0.17	20.6	26.1	0.14	21.2	26.3	20.584	0.17
				**mean**	**0.134**	**18.4**	**32.6**	**0.1**	**17.7**	**32.6**	**18.355**	**0.134**
				**n**	**2**	**2**	**2**	**2**	**2**	**2**	**2**	**2**
				**SEM**	**0.026**	**1.6**	**4.64**	**0.028**	**2.5**	**4.5**	**1.6**	**0.026**

Supplementary Table S6-continued

GIRK2		WTM model, μ=0.44								Free μ, c=0.03	
		Individual cells			Groups			Summary		Individuals cells	
Exp. #	See Fig.	*c*	*K*d (µM)	*I*max (µA)	*c*	*K*d (µM)	I_{max} (µA)	*K*d (µM)	*c*	μ	*K*d (µM)
1	3	0.024	7.4	Po=0.196				7.4	0.024	0.44	7.4
2					0.039	26.4	9.2	26.4	0.039		
3		0	2.7	8.4				2.8	0	fit unstable	
4	4a-c	0.03	18.5	25.6	0.029	12.6	21.4	12.6	0.029	0.62	8.2
5	Supp. 5				0.006	9.6	20	9.6	0.006		
6		0.07	11.2	3.5	0	11.3	5.5	11.2	0.07	fit unstable	
	**mean**	**0.03**	**10**	**12.5**	**0.019**	**15.0**	**14.05**	**11.3**	**0.028**	**0.53**	**7.8**
	**n**	**4**	**4**	**3**	**4**	**4**	**4**	**6**	**6**	**2**	**2**
	**SEM**	**0.013**	**3.1**	**5.5**	**0.008**	**3.3**	**3.42**	**3.1**	**0.009**	**0.06**	**0.3**
**GIRK1/2**		**WTM model, μ=0.44**									
4	4a-c	0.23	3.3	16.2	0.22	3.1	15.2	3.3	0.23		
7	4d	0.19	0.8	21.9	0.17	2.5	24.9	0.8	0.19		
8		0.34	2.4	13.1	0.31	2.2	13.6	2.4	0.34		
	**mean**	**0.25**	**2.2**	17.1	**0.23**	**2.6**	**17.9**	**2.2**	**0.25**		
	**n**	**3**	**3**	**3**	**3**	**3**	**3**	**3**	**3**		
	**SEM**	**0.04**	**0.6**	**2.1**	**0.03**	**0.2**	**2.9**	**0.6**	**0.04**		
**$GIRK1_{\Delta dCT}$/GIRK2**		**WTM model, μ=0.44**									
7	4d	0.09	7.5	41.3	0.06	6.6	41.1	7.5	0.09		
8		0.15	8.6	26.5	0.095	8.7	26.9	8.6	0.15		
	**mean**	**0.12**	**8.0**	**33.9**	**0.078**	**7.6**	**34**	**8.0**	**0.12**		
	**n**	**2**	**2**	**2**	**2**	**2**	**2**	**2**	**2**		
	**SEM**	**0.02**	**0.4**	**5.2**	**0.012**	**0.8**	**5**	**0.4**	**0.02**		

Supplementary Table 6. Experimental details and the WTM model fit parameters for all Gβγ dose-dependence experiments. I_{max} is the fitted maximal current. Cooperativity factor μ was fixed at 0.3 or 0.44. Fits with free μ (with fixed *c*=0.03) were also performed for GIRK2 data, but stable fits were obtained only in two experiments. GIRK2 channel surface density in experiments #2-6 was calculated from I_{max} (fit with μ=0.3) assuming $P_{o,max}$=0.19. In experiment #1 whole-cell currents in the 5 ng Gβ RNA group were 640±136 nA (n=8), corresponding to 0.91 μm^{-2} channel density. All currents in whole-cell mode were recorded in 24 mM $[K]_{out}$ solution. Data are presented for individual cells and groups (where available). In all experiments we measured the surface density of $G\beta\cdot_{YFP}G\gamma$ except #5, where we monitored relative changes in PM-attached Gβ in GMPs (instead of YFP-Gγ) and assumed Gβ density of 40 μm^{-2} with 5 ng Gβ RNA (from Fig. 3). In experiment #2, surface expression of $GIRK2_{HA}$ was measured in groups of cells expressing the different concentration of Gβγ (see Supplementary Methods), and currents were corrected for changes in channel expression.

Supplementary Table 7. p values for pull-down experiments

	p	q	DF
G1NdCT vs. G1NC	0.0001	4.820	51
G1NdCT vs. G1NCΔC1	0.3787	1.913	51
G1NdCT vs. G1NCΔC2	0.0189	3.215	51
G1NdCT vs. G1NCΔC3	0.0134	3.337	51
G1NdCT vs. G1CT	<0.0001	7.222	51
G1NdCT vs. G1(1-40)dCT	<0.0001	6.532	51
G1NdCT vs. G1(40-84)dCT	0.9632	0.9404	51
G1NdCT vs. Sumo dCT	<0.0001	6.853	51
G1NdCT vs. Sumo NT	<0.0001	7.418	51
	p	q	DF
G1NC vs. G1NCΔC1	0.7085	1.389	51
G1NC vs. G1NCΔC2	0.9752	0.8313	51
G1NC vs. G1NCΔC3	0.9998	0.4212	51
G1NC vs. G1CT	0.4784	1.707	51
G1NC vs. G1NdCT	0.0001	4.820	51
G1NC vs. G1(1-40)dCT	0.0932	2.562	51
G1NC vs. G1(40-84)dCT	0.0744	2.659	51
G1NC vs. Sumo dCT	0.1012	2.527	51
G1NC vs. Sumo NT	0.0109	3.389	51

Supplementary Table 7; for Fig. 7: Statistical comparison of Gβγ binding in constructs containing different regions of GIRK1 from pull-down experiments. The table presents p-values (p), t-values (q), and degrees of freedom (DF) for pairwise comparisons using Dunnett's test. G1NC or G1NdCT were used as controls in separate analyses to compare all other samples against them.

Supplementary Table 8. K_d and Hill coefficients from fits to Hill equation for dose-response relations from excised patch measurements.

channel	Gβγ type	preparation	K_d (nM)	n_H	reference
I_{KACh} (GIRK1/4)	$G\beta_1$ or $G\beta_2$ with $G\gamma_2$, $G\gamma_5$ or $G\gamma_7$	atrial myocyte	4-11	1.5	[31]
GIRK1/3	Bovine brain	*Xenopus* oocyte	11	1.5	[36]
GIRK 1/4	Bovine brain	*Xenopus* oocyte	10	1.5	
neuronal (GIRK 1/2?)	$G\beta_1\gamma_2$	locus coeruleus neuron	3.78	2.03	[37]
GIRK1/2	$G\beta_1\gamma_2$	*Xenopus* oocyte	11	1.37-2.04	ref. [23] and Fig. S11
GIRK 1/5 (GIRK5 of *Xenopus*)	$G\beta_1\gamma_2$	*Xenopus* oocyte	1.8-4.75	1.21-1.31	[38, 39]
I_{KACh} (GIRK1/4)	Bovine brain	atrial myocyte	6	3.12	[40]

Supplementary Table 9. Proteins constructs used in this work

Construct name	DNA in vector	Species	Remarks	Accession number
GIRK1	pGEM-HJ	rat		NP_113798.1
YFP-GIRK1	pGEM-HJ	rat	YFP in NT	
GIRK1Δ123(dCT)	pGEM-HJ	rat	GIRK1 (1-378)	
G1NC WT	pMXT	rat	GIRK1 NT(1-84)-Linker (QSTASQST)-CT(185-501)	
G1NCΔdCT	pMXT	rat	GIRK1 NT(1-84)-Linker (QSTASQST)-CT(184-380)	
G1NdCT	pMXT	rat	GIRK1 NT(1-84)-Linker (QSTASQST)-dCT(381-501)	
G1NCΔC1	pMXT	rat	GIRK1 NT(1-84)-Linker (QSTASQST)-CT(254-501)	
G1NCΔC2	pMXT	rat	GIRK1 NT(1-84)-Linker (QSTASQST)-CT(184-253, 321-501)	
G1NCΔC3	pMXT	rat	GIRK1 NT(1-84)-Linker (QSTASQST)-CT(184-319, 371-501)	
G1N(1-40)dCT	pMXT	rat	GIRK1 NT(1-40)-Linker (QSTASQST)-dCT(381-501)	
G1N(40-84)dCT	pMXT	rat	GIRK1 NT(40-84)-Linker (QSTASQST)-dCT(381-501)	
Sumo-G1NT	pGEM-HJ	rat	Sumo-GIRK1 NT(1-84)	
G1CT	pMXT	rat	GIRK1 CT(184-501)	
Sumo-G1dCT	pMXT	rat	Sumo-GIRK1 dCT(381-501)	
GIRK2	pGEM-HJ	mouse	GIRK2A, 414 a.a.	NP_001020755.1
G2NC	PGBXW	mouse	GIRK2 NT(1-95) Linker (QSTASQST) CT(194-414)	
$GIRK2_{trunc}$	pGEM-HJ	mouse	52-380 a.a	
$G2NC_{trunc}$	pGEM-HJ	mouse	GIRK2 NT(52-95) Linker (QSTASQST) CT(194-380)	
GIRK2-HA	pGEM-HJ	mouse	HA tag in P-loop --MDHI-HA-EDPS--	
GIRK2-CFP	pGEM-HJ	mouse	CFP in CT	
M2R	pGES	human		NP_001006631.1
IRK1-YFP, IRK1-CFP	pGSB	mouse	YFP or CFP in CT	NP_032451.1
Gβ1 wt	pGEM-HE	bovine		NP_786971.2
Myr-Gβ1	pGEM-HJ	bovine	myristoylated Gβ1	
YFP-Gβ1	pGEM-HJ	Bovine	YFP in NT with a Lys-Ser linker	
Split Venus2-Gβ1	pGEM-HJ	human	Split Venus2 in NT	
Gβ1-6Gly-YFP	pGEM-HJ	bovine	YFP in CT, fused to Gβ1 by a linker of GGGGGG (6 glycines)	
continued on next page				

CFP-2Gly-Gβ1	pGEM-HJ	bovine	YFP in NT, fused to Gβ by a linker of GG (2 glycines)	
Gγ_2	pGEM-HJ	bovine		P63212.2
Gγ_2 C68S	pGEM-HE	bovine		
Split Venus1-Gγ2	pGEM-HJ	bovine	Split Venus1 in NT	
YFP-Gγ_2, YFP-Gγ_{2C68S}	pMXT	bovine	YFP in NT	
Gα_{i3}	pGEM-HJ	human		NP_006487.1
Phosducin	pGEM-HE	bovine	myristoylated phosducin	NP_002588.3

DNA constructs encoding mouse GIRK2 (GIRK2A, 414 a.a.), GIRK2$_{HA}$, C-terminally CFP labeled GIRK2 (GIRK2-CFP), bovine Gβ_1, bovine Gγ_2, human m2R, rat GIRK1, N-terminally YFP labeled GIRK1 (YFP-GIRK1), N-terminally YFP labeled Gγ_2 (YFP-Gγ_2), N-terminally YFP labeled Gβ_1 (YFP-Gβ, with a Lys-Ser linker), G1NC (the cytosolic N-a.a. 1-84 and C-termini a.a 183-501 of GIRK1 connected by an 8-a.a. linker, QSTASQST), G2NC (the full cytosolic N- a.a. 1-95 and C-termini a.a. 194-404 of GIRK2 connected by a 2-a.a. linker, Lys-Leu), full length human Gα_{i3} and myristoylated bovine phosducin were described previously[10, 41-43]. TEVC, two-electrode whole-cell voltage clamp.

Supplementary Table 10. Antibodies used in this study

Antibody	SOURCE	Dilution	IDENTIFIER
Donkey IgG	Jackson ImmunoResearch Labs	1:200	Cat. No. 017-000-003; RRID:AB_2337256; Lot 131795
Rabbit polyclonal anti-Gβ (T-20)	Santa Cruz Biotechnology	1:200 (GMP) 1:300 - 1:400 (WB)	Cat. No. sc-378; RRID:AB_631542; currently discontinued
Rabbit polyclonal GNB1 ($G\beta_1$)	GeneTex	1:200 (GMP) 1:1000 (WB, peptide array)	GTX114442; RRID:AB_10619473; Lot 43565
Rabbit polyclonal GIRK1	Alomone	1:300	#APC-005; RRID: AB_2040113
Rabbit polyclonal GFP	Abcam	1:1000	AB6556; RRID:AB_305564
Goat Anti-Rabbit IgG H&L–DyLight650	Abcam	1:300	ab96886; RRID:AB_10680254; Lot GR3228258-6
Anti-His_6-Peroxidase mouse monoclonal, clone BMG-His-1	Roche	1:500	Cat. No. 11 965 085 001; RRID: AB_514487
Goat Anti-Rabbit IgG, (H+L) HRP conjugated	Jackson ImmunoResearch Labs	1:40000	Cat. No. 111-035-144, RRID:AB_2307391

Supplementary Table 11. Molecular dynamics stimulations: system parameters and AphaFold3 per-residue pLDDT values from AlphaFold-predicted structures

Molecular dynamics stimulations: system parameters						
	**G1NC (CG)**	**$G1NC_{\Delta dCT}$ (CG)**	**G2NC (CG)**	**$G2NC_{trunc}$ (CG)**	**G1NC+membr (CG)**	**G1NC (AA)**
**Box dimensions**	201.489 201.489 201.489	185.502 185.502 185.502	188.897 188.897 188.897	170.032 170.032 170.032	359.613 359.613 215.749	203.276 203.276 213.469
**Total number of atoms**	181385	162806	168814	122296	635056	866735
**Number of water molecules**	57051	51354	53276	38178	190036	271835
**Salt concentration**	140.64 mmol Na^+ 141.94 mmol Cl^-	147.60 mmol Na^+ 140.40 mmol Cl^-	116.95 mmol Na^+ 96.12 mmol Cl^-	111.86 mmol Na^+ 96.37 mmol Cl^-	140.11 mmol Na^+ 138.95 mmol Cl^-	108.34 mmol Na^+ 108.34 mmol Cl^-
**Membrane**	No	No	No	No	Yes	No
**Number of membrane lipids**	/	/	/	/	3976 POPC	/
**Production Runs and Time**	5 x 5 µs	5 x 5 µs	10 x 5 µs	10 x 5 µs	3 x 5 µs	3 x 500 ns

Average AlphaFold pLDDT values grouped by protein segments in the different systems				
	**G1NC (CG)**	**$G1NC_{\Delta dCT}$**	**G2NC**	**$G2NC_{trunc}$**
**NT**	49.16	50.57	57.68	73.54
**CT-core**	81.62	81.44	91.03	91.06
**dCT**	29.46	-	38.3	-
**Gβγ**	88.34	90.65	91.16	90.12

AA, all-atom; CG, coarse grain; pLDDT, predicted local distance difference test. The levels of confidence in AlphaFold models is defined as: 70 – 90: confident; 50-70: low-confidence; <50: very low confidence.

Supplementary Table 12. Binding of Gγ$_{prenyl}$ to G1NC and G2NC.

The results are from 5 µs production runs. The % interaction shown stands for percentage of frames with at least one prenylation tail (Gγ$_{prenyl}$) bound to the channel. For details of interactions of individual subunits, see Supplementary Table 14.

	**% interaction**			
Run #	G1NC	Truncated G1NC (G1NC$_{ΔdCT}$)	G2NC	Truncated G2NC
1	100%	0.09%	7.48%	4.72%
2	100%	0.29%	6.60%	0.02%
3	100%	0.35%	8.15%	13.26%
4	100%	0.05%	0.01%	0.15%
5	100%	0.01%	1.55%	0.25%
6			13.30%	10.72%
7			4.66%	5.15%
8			12.38%	0.12%
9			5.51%	18.33%
10			4.17%	0.09%
**Mean± SEM**	**100±0%**	**0.16±0.07%**	**6,38% ± 1,34%**	**5,28% ± 2,1%**
**Statistics**	p=0.0079 between G1NC and G1NC$_{ΔdCT}$, Mann-Whitney test		p=0.48 between G2NC and G2NC$_{trunc}$, Mann-Whitney test	
	**Kruskal-Wallis test followed by Dunn's multiple comparison vs. G1NC**			
		p=0.0003	p=0.039	p= 0.0065

Supplementary Table 13. Binding of $G\gamma_{prenyl}$ to the hydrophobic pocket at Gβ – G1NC and Gβ – G2NC systems.

The results are from 5 µs production runs. Binding of the tail to Gβ is shown for a.a. 314-340 of Gβ (the last 27 C-terminal a.a.), where the interaction took place. The % interaction shown stands for percentage of frames with prenylation tail ($G\gamma_{prenyl}$) bound to Gβ. Statistical analysis was done with two-tailed T-test of Mann-Whitney test.

The Gβ-G1NC system

	**Non-truncated G1NC**		**Truncated G1NC ($G1NC_{\Delta dCT}$)**	
Run #	% interaction	Residues in Gβ involved	% interaction	Residues in Gβ involved
1	0	/	32.79%	W339 (99.9%), K337 (0.1%)
2	0%	/	34.59%	W339 (99.9%), K337, T239 (0.1%)
3	0.16%	W339 (100%)	30.88%	W339 (99.2%), F335 (0.8%)
4	0%	/	93.27%	W339 (99.9%), F335 (0.1%)
5	0%	/	80.55%	W339 (100%)
**Mean± SEM**	**0.03% ± 0.03%**		**54.42% ± 13.43%**	p=0.0079 Mann-Whitney test (compared to non-truncated)

The Gβ-G2NC system

	**Non-truncated G2NC**		**Truncated G2NC**	
Run #	% interaction	Residues in Gβ involved	% interaction	Residues in Gβ involved
1	83.44%	W339 (100%)	69.62%	W339 (97.4%), K337 (1.5%), F335 (0.6%), T329 (0.5%)
2	4.98%	W339 (83.8%), F335 (15.1%), K337 (1.1%)	95.50%	W339 (100%)
3	6.18%	W339 (99.9%), K337 (0.1%)	92.74%	W339 (100%)
4	87.46%	W339 (100%)	81.95%	W339 (100%)
5	32.49%	W339 (99.7%), K337 (0.3%)	16.63%	W339 (99.9%), T329 (0.1%)
6	80.81%	W339 (100%)	96.26%	W339 (100%)
7	76.65%	W339 (97.1%), F335 (1.7%), K337 (1.2%)	54.02%	W339 (86.5%), K337 (9.6%), F335 (3.9%)
8	14.16%	W339 (97.9%), K337 (1.3%), F335 (0.8%)	53.62%	W339 (99.8%), T329 (0.2%)
9	70.01%	W339 (96.5%), F335 (3.1%), K337 (0.3%)	40.08%	W339 (100%)
10	40.02%	W339 (98.7%), F335 (0.8%), K337 (0.6%)	71.88%	W339 (99.8%), K337 (0.1%), T329 (0.1%)
**Mean± SEM**	**49,62% ± 10,66%**		**67,23% ± 8,3%**	p=0.2096 non-paired t-test compared to non-truncated

The interaction of Gβ with $G\gamma_{prenyl}$ was significantly greater in G2NC than in G1NC (p=0.0417, Kruskal-Wallis test with Dunn's multiple comparisons test.

Supplementary Table 14. MD simulation analysis details

List of sub-tables

Note: "Prenylation Tail" stands for $G\gamma_{prenyl}$

Table

Sub-Table 1: G1NC - Prenylation Tai and NT-dCT binding

Table 2: Truncated G1NC - Prenylation Tail binding

Sub-Table 3: G2NC - Prenylation Tail binding

Sub-Table 4: Truncated G2NC - Prenylation Tail binding

Sub-Table 5: G1NC with Membrane - Prenylation Tail binding

Sub-Table 6: G1NC - all-atom simulations - Prenylation Tail binding

Sub-Sub-Table 1: G1NC - Prenylation Tail and NT-dCT binding

Run	Simulation time	G1NC – Percentage of frames with min. 1 bound prenylation tail	G1NC –Average of each subunit	Gβγ – Percentage of frames with min. 1 bound prenylation tail	Gβγ – Prenylation tail binding [average percentages for each subunit]	G1NC - NT-dCT Interactions [Percentage of frames with min. 1 interaction NT-dCT]	G1NC - NT-dCT Interactions [average percentages for each subunit]
**Run 1**	5 µs	100%	99.89%	93.58%	47.65%	99.79%	63.58%
**Run 2**	5 µs	100%	99.85%	99.93%	76.94%	99.86%	70.55%
**Run 3**	5 µs	100%	99.44%	98.28%	54.83%	99.51%	54.71%
**Run 4**	5 µs	100%	99.97%	90.85%	41.09%	97.24%	54.51%
**Run 5**	5 µs	100%	100%	41.89%	13.05%	98.29%	54.87%
**Mean ± SEM**		**100% ± 0%**	**99.83% ± 0.1%**	**84.91% ± 10.88%**	**46.71% ± 10.36%**	**98.94% ± 0.51%**	**59.64% ± 3.22%**

Run	Simulation Time	Chain	G1NC - Tail binding	Gβγ - Tail binding	NT - dCT Interactions
**Run 1**	5 µs	Chain A	99.91%	36.34%	62.77%
		Chain B	99.98%	31.95%	91.95%
		Chain C	99.99%	76.11%	2.70%
		Chain D	99.68%	46.18%	96.90%
		**Mean ± SEM**	**99.89% ± 0.07%**	**47.65% ± 9.94%**	**63.58% ± 21.65%**
**Run 2**	5 µs	Chain A	99.93%	54.68%	60.44%
		Chain B	99.98%	92.48%	98.64%
		Chain C	99.99%	88.08%	82.09%
		Chain D	99.48%	72.51%	41.02%
		**Mean ± SEM**	**99,84% ± 0,12%**	**76,94% ± 8,57%**	**70,55% ± 12,57%**
**Run 3**	5 µs	Chain A	99.93%	21.15%	6.56%
		Chain B	99.74%	86.75%	95.30%
		Chain C	99.79%	50.22%	65.93%
		Chain D	98.29%	61.21%	51.04%
		**Mean ± SEM**	**99,44% ± 0,38%**	**54,83% ± 13,59%**	**54,71% ± 18,5%**
**Run 4**	5 µs	Chain A	99.99%	62.39%	76.89%
		Chain B	99.87%	49.16%	74.89%
		Chain C	100.00%	3.01%	36.57%
		Chain D	100.00%	49.78%	29.69%
		**Mean ± SEM**	**99,96% ± 0,03%**	**41,08% ± 13,05%**	**54,51% ± 12,43%**
**Run 5**	5 µs	Chain A	99.99%	4.31%	78.24%
		Chain B	100.00%	11.84%	27.97%
		Chain C	100.00%	28.77%	81.48%
		Chain D	100.00%	7.29%	31.80%
		**Mean ± SEM**	**100% ± 0%**	**13,05% ± 5,46%**	**54,87% ± 14,46%**

Supplementary Table 14 continued

Sub-Table 2: Truncated G1NC - Prenylation Tail binding

Run	Simulation time	Trunc. G1NC – Percentage of frames with min. 1 bound prenylation tail	Trunc. G1NC – Prenylation tail binding [Average of each subunit]	Gβγ – Prenylation tail last atom biinding - Percentage of frames with min. 1 bound prenylation tail	Gβγ – Prenylation tail binding [average percentages for each subunit]
**Run 1**	5 µs	0.09%	0.02%	100.00%	92.01%
**Run 2**	5 µs	0.29%	0.09%	99.99%	89.87%
**Run 3**	5 µs	0.35%	0.09%	100.00%	92.96%
**Run 4**	5 µs	0.05%	0.01%	100.00%	95.99%
**Run 5**	5 µs	0.01%	0%	100.00%	93.35%
**Mean ± SEM**		**0.16% ± 0.07%**	**0.04% ± 0.02%**	**100% ± 0%**	**92.84% ± 0.99%**

Run	Simulation 1	Chain	Trunc. G1NC - Tail bi	Gβγ - Tail binding
**Run 1**	5 µs	Chain A	0.00%	94.01%
		Chain B	0.00%	97.94%
		Chain C	0.08%	88.09%
		Chain D	0.00%	87.98%
		**Mean ± SEM**	**0,02% ± 0,02%**	**92,01% ± 2,43%**
**Run 2**	5 µs	Chain A	0.00%	85.37%
		Chain B	0.00%	96.20%
		Chain C	0.27%	87.67%
		Chain D	0.10%	90.22%
		**Mean ± SEM**	**0,09% ± 0,06%**	**89,86% ± 2,33%**
**Run 3**	5 µs	Chain A	0.27%	94.32%
		Chain B	0.08%	89.88%
		Chain C	0.00%	91.04%
		Chain D	0.00%	96.59%
		**Mean ± SEM**	**0,09% ± 0,06%**	**92,96% ± 1,53%**
**Run 4**	5 µs	Chain A	0.00%	95.54%
		Chain B	0.02%	97.18%
		Chain C	0.02%	93.59%
		Chain D	0.01%	97.65%
		**Mean ± SEM**	**0,01% ± 0%**	**95,99% ± 0,92%**
**Run 5**	5 µs	Chain A	0.00%	96.19%
		Chain B	0.00%	94.82%
		Chain C	0.01%	95.55%
		Chain D	0.00%	86.84%
		**Mean ± SEM**	**0% ± 0%**	**93,35% ± 2,19%**

Sub-Table 3: G2NC - Prenylation Tail binding

Run	Simulation time	G2NC – Prenylation tail binding [Percentage of frames with min. 1 bound prenylation tail]	G2NC – Prenylation tail binding [Average of each subunit]	Gβγ – Prenylation tail (last atom) binding [Percentage of frames with min. 1 bound prenylation tail]
Run 1	5 µs	7.48%	1.78%	100.00%
Run 2	5 µs	6.60%	1.67%	99.96%
Run 3	5 µs	8.15%	2.05%	100.00%
Run 4	5 µs	0.01%	0.00%	99.99%
Run 5	5 µs	1.55%	0.39%	100.00%
Run 6	5 µs	13.30%	3.33%	99.99%
Run 7	5 µs	4.66%	1.69%	99.99%
Run 8	5 µs	12.38%	3.12%	100.00%
Run 9	5 µs	5.51%	2.18%	99.97%
Run 10	5 µs	4.17%	1.36%	99.97%
Mean ± SEM		6,38% ± 1,34%	1,76% ± 0,33%	99,99% ± 0%

Run	Simulation Time	Chain	G2NC - Tail binding	Gβγ - Tail binding
Run 1	5 µs	Chain A	0.00%	82.50%
		Chain B	0.00%	95.95%
		Chain C	7.47%	93.41%
		Chain D	0.02%	91.72%
		**Mean ± SEM**	**1,87% ± 1,87%**	**90,9% ± 2,93%**
Run 2	5 µs	Chain A	0.00%	81.13%
		Chain B	1.27%	86.16%
		Chain C	0.10%	85.54%
		Chain D	5.30%	96.17%
		**Mean ± SEM**	**1,67% ± 1,24%**	**87,25% ± 3,18%**
Run 3	5 µs	Chain A	0.00%	91.32%
		Chain B	8.10%	83.69%
		Chain C	0.06%	94.30%
		Chain D	0.02%	84.88%
		**Mean ± SEM**	**2,05% ± 2,02%**	**88,55% ± 2,55%**
Run 4	5 µs	Chain A	0.00%	98.74%
		Chain B	0.01%	89.11%
		Chain C	0.00%	94.81%
		Chain D	0.00%	97.86%
		**Mean ± SEM**	**0% ± 0%**	**95,13% ± 2,18%**
Run 5	5 µs	Chain A	0.02%	91.31%
		Chain B	0.01%	82.21%
		Chain C	0.04%	92.49%
		Chain D	1.49%	95.55%
		**Mean ± SEM**	**0,39% ± 0,37%**	**90,39% ± 2,87%**
Run 6	5 µs	Chain A	0.04%	91.37%
		Chain B	0.05%	92.85%
		Chain C	0.01%	92.82%
		Chain D	13.21%	93.60%
		**Mean ± SEM**	**3,33% ± 3,29%**	**92,66% ± 0,47%**
Run 7	5 µs	Chain A	0.21%	93.78%
		Chain B	0.02%	95.85%
		Chain C	2.39%	75.78%
		Chain D	4.14%	91.46%
		**Mean ± SEM**	**1,69% ± 0,98%**	**89,22% ± 4,57%**
Run 8	5 µs	Chain A	0.01%	93.72%
		Chain B	0.22%	82.15%
		Chain C	12.16%	93.75%
		Chain D	0.09%	87.05%
		**Mean ± SEM**	**3,12% ± 3,01%**	**89,17% ± 2,82%**
Run 9	5 µs	Chain A	0.01%	93.59%
		Chain B	0.03%	91.12%
		Chain C	4.90%	94.36%
		Chain D	3.77%	83.03%
		**Mean ± SEM**	**2,18% ± 1,27%**	**90,52% ± 2,59%**
Run 10	5 µs	Chain A	0.25%	84.88%
		Chain B	0.01%	92.24%
		Chain C	3.67%	93.44%
		Chain D	1.50%	79.48%
		**Mean ± SEM**	**1,36% ± 0,84%**	**87,51% ± 3,28%**

Supplementary Table 14 continued. Sub-Table 3: G2NC - Prenylation Tail binding

Run	Simulation time	G2NC – Prenylation tail binding [Percentage of frames with min. 1 bound prenylation tail]	G2NC – Prenylation tail binding [Average of each subunit]	Gβγ – Prenylation tail (last atom) binding [Percentage of frames with min. 1 bound prenylation tail]
Run 1	5 µs	7.48%	1.78%	100.00%
Run 2	5 µs	6.60%	1.67%	99.96%
Run 3	5 µs	8.15%	2.05%	100.00%
Run 4	5 µs	0.01%	0.00%	99.99%
Run 5	5 µs	1.55%	0.39%	100.00%
Run 6	5 µs	13.30%	3.33%	99.99%
Run 7	5 µs	4.66%	1.69%	99.99%
Run 8	5 µs	12.38%	3.12%	100.00%
Run 9	5 µs	5.51%	2.18%	99.97%
Run 10	5 µs	4.17%	1.36%	99.97%
**Mean ± SEM**		**6,38% ± 1,34%**	**1,76% ± 0,33%**	**99,99% ± 0%**

Run	Simulation Time	Chain	G2NC - Tail binding	Gβγ - Tail binding
Run 1	5 µs	Chain A	0.00%	82.50%
		Chain B	0.00%	95.95%
		Chain C	7.47%	93.41%
		Chain D	0.02%	91.72%
		**Mean ± SEM**	**1,87% ± 1,87%**	**90,9% ± 2,93%**
Run 2	5 µs	Chain A	0.00%	81.13%
		Chain B	1.27%	86.16%
		Chain C	0.10%	85.54%
		Chain D	5.30%	96.17%
		**Mean ± SEM**	**1,67% ± 1,24%**	**87,25% ± 3,18%**
Run 3	5 µs	Chain A	0.00%	91.32%
		Chain B	8.10%	83.69%
		Chain C	0.06%	94.30%
		Chain D	0.02%	84.88%
		**Mean ± SEM**	**2,05% ± 2,02%**	**88,55% ± 2,55%**
Run 4	5 µs	Chain A	0.00%	98.74%
		Chain B	0.01%	89.11%
		Chain C	0.00%	94.81%
		Chain D	0.00%	97.86%
		**Mean ± SEM**	**0% ± 0%**	**95,13% ± 2,18%**
Run 5	5 µs	Chain A	0.02%	91.31%
		Chain B	0.01%	82.21%
		Chain C	0.04%	92.49%
		Chain D	1.49%	95.55%
		**Mean ± SEM**	**0,39% ± 0,37%**	**90,39% ± 2,87%**
Run 6	5 µs	Chain A	0.04%	91.37%
		Chain B	0.05%	92.85%
		Chain C	0.01%	92.82%
		Chain D	13.21%	93.60%
		**Mean ± SEM**	**3,33% ± 3,29%**	**92,66% ± 0,47%**
Run 7	5 µs	Chain A	0.21%	93.78%
		Chain B	0.02%	95.85%
		Chain C	2.39%	75.78%
		Chain D	4.14%	91.46%
		**Mean ± SEM**	**1,69% ± 0,98%**	**89,22% ± 4,57%**
Run 8	5 µs	Chain A	0.01%	93.72%
		Chain B	0.22%	82.15%
		Chain C	12.16%	93.75%
		Chain D	0.09%	87.05%
		**Mean ± SEM**	**3,12% ± 3,01%**	**89,17% ± 2,82%**
Run 9	5 µs	Chain A	0.01%	93.59%
		Chain B	0.03%	91.12%
		Chain C	4.90%	94.36%
		Chain D	3.77%	83.03%
		**Mean ± SEM**	**2,18% ± 1,27%**	**90,52% ± 2,59%**
Run 10	5 µs	Chain A	0.25%	84.88%
		Chain B	0.01%	92.24%
		Chain C	3.67%	93.44%
		Chain D	1.50%	79.48%
		**Mean ± SEM**	**1,36% ± 0,84%**	**87,51% ± 3,28%**

Supplementary Table 14 continued

Sub-Table 4: Truncated G2NC - Prenylation Tail binding

Run	Simulation time	Trunc. G2NC – Prenylation tail binding [Percentage of frames with min. 1 bound prenylation	Trunc. G2NC – Prenylation tail binding [Average of each subunit]	Gβγ – Prenylation tail (last atom) binding [Percentage of frames with min. 1 bound prenylation tail]	Gβγ – Prenylation tail binding [average percentages for each subunit]
**Run 1**	5 µs	4.72%	1.20%	100.00%	90.62%
**Run 2**	5 µs	0.02%	0.01%	100.00%	97.92%
**Run 3**	5 µs	13.26%	3.32%	100.00%	78.85%
**Run 4**	5 µs	0.15%	0.04%	99.99%	79.64%
**Run 5**	5 µs	0.25%	0.06%	99.99%	89.19%
**Run 6**	5 µs	10.72%	2.83%	100.00%	91.60%
**Run 7**	5 µs	5.15%	1.29%	99.98%	86.00%
**Run 8**	5 µs	0.12%	0.03%	99.90%	78.55%
**Run 9**	5 µs	18.33%	4.59%	100.00%	84.67%
**Run 10**	5 µs	0.09%	0.02%	100.00%	83.23%
**Mean ± SEM**		**5,28% ± 2,1%**	**1,34% ± 0,53%**	**99,99% ± 0,01%**	**86,03% ± 2%**

Run	Simulation Time	Chain	Trunc. G2NC - Tail binding	Gβγ - Tail binding
**Run 1**	5 µs	Chain A	3.16%	80.74%
		Chain B	0.00%	87.76%
		Chain C	0.04%	99.02%
		Chain D	1.59%	94.95%
		**Mean ± SEM**	**1,2% ± 0,75%**	**90,62% ± 4,03%**
**Run 2**	5 µs	Chain A	0.00%	95.89%
		Chain B	0.01%	95.07%
		Chain C	0.01%	98.94%
		Chain D	0.01%	99.24%
		**Mean ± SEM**	**0,01% ± 0%**	**97,28% ± 1,06%**
**Run 3**	5 µs	Chain A	13.22%	82.91%
		Chain B	0.03%	43.18%
		Chain C	0.00%	99.22%
		Chain D	0.01%	90.09%
		**Mean ± SEM**	**3,32% ± 3,3%**	**78,85% ± 12,35%**
**Run 4**	5 µs	Chain A	0.02%	95.94%
		Chain B	0.06%	91.62%
		Chain C	0.07%	95.98%
		Chain D	0.01%	34.28%
		**Mean ± SEM**	**0,04% ± 0,01%**	**79,46% ± 15,09%**
**Run 5**	5 µs	Chain A	0.09%	85.84%
		Chain B	0.16%	91.24%
		Chain C	0.00%	86.40%
		Chain D	0.00%	93.28%
		**Mean ± SEM**	**0,06% ± 0,04%**	**89,19% ± 1,82%**
**Run 6**	5 µs	Chain A	7.14%	88.92%
		Chain B	0.01%	99.44%
		Chain C	3.21%	95.20%
		Chain D	0.94%	82.82%
		**Mean ± SEM**	**2,82% ± 1,59%**	**91,6% ± 3,64%**
**Run 7**	5 µs	Chain A	5.08%	77.60%
		Chain B	0.00%	88.80%
		Chain C	0.01%	93.02%
		Chain D	0.07%	84.57%
		**Mean ± SEM**	**1,29% ± 1,26%**	**86% ± 3,29%**
**Run 8**	5 µs	Chain A	0.07%	67.77%
		Chain B	0.05%	68.19%
		Chain C	0.00%	83.40%
		Chain D	0.00%	94.83%
		**Mean ± SEM**	**0,03% ± 0,02%**	**78,55% ± 6,53%**
**Run 9**	5 µs	Chain A	0.00%	89.17%
		Chain B	18.27%	90.91%
		Chain C	0.07%	84.27%
		Chain D	0.02%	74.31%
		**Mean ± SEM**	**4,59% ± 4,56%**	**84,66% ± 3,73%**
**Run 10**	5 µs	Chain A	0.01%	99.40%
		Chain B	0.00%	84.62%
		Chain C	0.01%	81.62%
		Chain D	0.07%	67.30%
		**Mean ± SEM**	**0,02% ± 0,02%**	**83,23% ± 6,58%**

Supplementary Table 14 continued

Sub-Table 5: G1NC with Membrane - Prenylation Tail binding

Run	Simulation time	G1NC – Prenylation tail binding [Percentage of frames with min. 1 bound prenylation tail]	G1NC – Prenylation tail binding [Average of each subunit]	Gβγ – Prenylation tail (last atom) binding [Percentage of frames with min. 1 bound prenylation tail]	Gβγ – Prenylation tail binding [average percentages for each subunit]
**Run 1**	5 μs	100%	68.30%	99.53%	41.88%
**Run 2**	5 μs	100%	66.66%	99.31%	37.52%
**Run 3**	5 μs	100%	66.77%	99.91%	59.19%
**Mean ± SEM**		**100% ± 0%**	**67,24% ± 0,53%**	**99,58% ± 0,18%**	**46,2% ± 6,62%**

Run	Simulation time	Prenylation tail - Membrane binding [Percentage of frames with min. 1 bound prenylation tail]	Prenylation tail - Membrane binding [average percentages for each subunit]	Prenylation tail - Membrane binding (start 2 μs) [Percentage of frames with min. 1 bound prenylation tail]
**Run 1**	5 μs	63.29%	10.55%	100.00%
**Run 2**	5 μs	0.05%	0.01%	0.09%
**Run 3**	5 μs	0.01%	0.00%	0.01%
**Mean ± SEM**		**21,12% ± 21,09%**	**3,52% ± 3,52%**	**33,37% ± 33,32%**

Run	Simulation Time	Chain	G1NC - Tail binding	Gβγ - Tail binding	Prenylation tail - Membrane
**Run 1**	5 μs	Chain A	100.00%	18.08%	0.00%
		Chain B	100.00%	85.41%	0.00%
		Chain C	100.00%	61.58%	0.00%
		Chain D	100.00%	2.46%	0.00%
		Chain E	0.00%	33.39%	63.29%
		Chain F	9.79%	96.56%	0.01%
		**Mean ± SEM**	**68,3% ± 20,09%**	**49,58% ± 15,39%**	**10,55% ± 10,55%**
**Run 2**	5 μs	Chain A	99.96%	46.10%	0.05%
		Chain B	100.00%	0.00%	0.00%
		Chain C	100.00%	0.06%	0.00%
		Chain D	100.00%	0.02%	0.00%
		Chain E	0.00%	85.43%	0.00%
		Chain F	0.00%	93.48%	0.00%
		**Mean ± SEM**	**66,66% ± 21,08%**	**37,52% ± 18%**	**0,01% ± 0,01%**
**Run 3**	5 μs	Chain A	100.00%	56.24%	0.00%
		Chain B	99.99%	24.58%	0.00%
		Chain C	99.47%	71.91%	0.01%
		Chain D	98.66%	12.86%	0.00%
		Chain E	2.24%	96.36%	0.00%
		Chain F	0.00%	93.19%	0.00%
		**Mean ± SEM**	**66,73% ± 20,75%**	**59,19% ± 14,2%**	**0% ± 0%**

Supplementary Table 14 continued

Sub-Table 6: G1NC - all-atom simulations - Prenylation Tail binding

Run	Simulation time	G1NC – Prenylation tail binding [Percentage of frames with min. 1 bound prenylation tail]	G1NC – Prenylation tail binding [Average of each subunit]	Gβγ – Prenylation tail (last atom) binding [Percentage of frames with min. 1 bound prenylation tail]	Gβγ – Prenylation tail binding [average percentages for each subunit]	NT-dCT Interactions [Percentage of frames with min. 1 interaction NT-dCT]	NT-dCT Interactions [Percentage of frames with min. 1 bound prenylation tail]
**Run 1**	500 ns	100%	99.90%	95.26%	66.68%	98.99%	52.88%
**Run 2**	450 ns	100%	99.14%	94.09%	42.32%	100.00%	71.57%
**Run 3**	500 ns	100%	98.61%	91.89%	44.10%	97.02%	51.68%
**Mean ± SEM**		**100% ± 0%**	**99,22% ± 0,37%**	**93,75% ± 0,99%**	**51,03% ± 7,84%**	**98,67% ± 0,88%**	**58,71% ± 6,44%**

Run	Simulation Time	Chain	G1NC-AA - Tail binding	Gβγ - Tail binding	NT-dCT Interactions
**Run 1**	500 ns	Chain A	100.00%	64.83%	98.34%
		Chain B	100.00%	63.43%	51.66%
		Chain C	100.00%	51.32%	38.51%
		Chain D	99.61%	87.15%	23.01%
		**Mean ± SEM**	**99,9% ± 0,1%**	**66,68% ± 7,47%**	**52,88% ± 16,25%**
**Run 2**	500 ns	Chain A	97.98%	72.49%	99.81%
		Chain B	100.00%	20.55%	14.14%
		Chain C	99.95%	61.60%	93.98%
		Chain D	98.63%	14.62%	73.88%
		**Mean ± SEM**	**99,14% ± 0,5%**	**42,32% ± 14,5%**	**70,45% ± 19,58%**
**Run 3**	500 ns	Chain A	100.00%	62.88%	84.42%
		Chain B	100.00%	16.65%	66.66%
		Chain C	99.79%	81.19%	11.83%
		Chain D	94.64%	15.69%	43.82%
		**Mean ± SEM**	**98,61% ± 1,32%**	**44,1% ± 16,56%**	**51,68% ± 15,67%**

Supplementary Table 15: Primers using for DNA cloning

SpV-Gβ

Forward primers:

TCCGAATTCTCTAGAATGGACAAGCAGAAG

CAAGATCTGGAACTAAAAGCTTGATCTGGTT

Reverse primers:

CTTCTGCTTGTCCATTCTAGAGAATTCGGA

AACCAGATCAAGCTTTTAGTTCCAGATCTTG

SpV-Gγ

Forward primers:

TCCGAATTCTCTAGAATGGTGAGCAAGGGC

CTGTGCTATCCTTTAAAAGCTTGATCTGGTT

Reverse primers:

GCCCTTGCTCACCATTCTAGAGAATTCGGA

AACCAGATCAAGCTTTTAAAGGATAGCACAG

G1NdCT:

Forward primers:

GACCGCTTCACATAGCACCGCGATCTGGTTAC

GCTAGCCAGTCTACGGAAAGACACAATTCTGTGGAG

Reverse primers:

GTAACCAGATCGCGGTGCTATGTGAAGCGGTCAG

CAGAATTGTGTCTTTCCGTAGACTGGCTAGCC

G1N(1-40)dCT

Forward primers:

CAGTCTACGGCTAGCCAGTCTACGGAAAGAC

CCCCTCCGTATTATGTCTGCACTCCGAAGGA

Reverse primers:

CCTTCGGAGTGCAGACATAATACGGAGGGG

AGCCGTAGACTGCTTGGGTACAAGCTGCTGC

G1N(40-84)dCT

Forward primers:

CAGTCTACGGCTAGCCAGTCTACGGAAAGAC

CCCCTCCGTATTATGAAGAAGAAACGGCAGCG

Reverse primers:

CGTTTCTTCTTCATAATACGGAGGGGAGATC

CAAGTGGCGTTGGAACCTCCAGTCTACGGCT

Sumo-G1NT

Forward primers:

GATATCCCAACGACCTCTGCACTCCGAAGGAA

GATCCGAATTCATGGGCAACGATCACATTAAC

Reverse primers:

GTGATCGTTGCCCATGAATTCGGATCCCCGGG

TCGGAGTGCAGAGGTCGTTGGGATATCATC

Sumo-G1dCT
Forward primers:
TCCCAACGACCGAAAGACACAATTCTGTGGAG
CCCTCCGTATTATGGGCAACGATCACATTAAC
Reverse primers:
TGGATCTCCCCTCCGTATTATGGGCAACGATC
GATGATATCCCAACGACCGAAAGACACAATT

YFP-Gγ(C68S)
Fwd: GAAGAAGTTTTTCAGTGCTATCCTTTAAG
Rev: CTTAAAGGATAGCACTGAAAAACTTCTTC

Supplementary references

1. Yakubovich D*, et al.* A quantitative model of the GIRK1/2 channel reveals that its basal and evoked activities are controlled by unequal stoichiometry of Gα and Gβγ. *PLoS Comput Biol* **11**, e1004598 (2015).

2. Berlin S*, et al.* A collision coupling model governs the activation of neuronal GIRK1/2 channels by muscarinic-2 receptors. *Front Pharmacol* **11**, 1216 (2020).

3. Dascal N, Kahanovitch U. The roles of Gβγ and Gα in gating and regulation of GIRK channels. *Int Rev Neurobiol* **123**, 27–85 (2015).

4. Marcoline FV, Furth J, Nayak S, Grabe M, Macey RI. Berkeley Madonna version 10-A simulation package for solving mathematical models. *CPT Pharmacometrics Syst Pharmacol* **11**, 290–301 (2022).

5. Ivanova-Nikolova TT, Nikolov EN, Hansen C, Robishaw JD. Muscarinic K^+ channel in the heart. Modal regulation by G protein βγ subunits. *J Gen Physiol* **112**, 199–210 (1998).

6. Sadja R, Alagem N, Reuveny E. Graded contribution of the Gβγ binding domains to GIRK channel activation. *Proc Natl Acad Sci U S A* **99**, 10783–10788 (2002).

7. Touhara KK, MacKinnon R. Molecular basis of signaling specificity between GIRK channels and GPCRs. *Elife* **7**, (2018).

8. Sarvazyan NA, Remmers AE, Neubig RR. Determinants of G_{i1}α and βγ binding. Measuring high affinity interactions in a lipid environment using flow cytometry. *J Biol Chem* **273**, 7934–7940 (1998).

9. Dessauer CW, Gilman AG. Purification and characterization of a soluble form of mammalian adenylyl cyclase. *J Biol Chem* **271**, 16967–16974 (1996).

10. Kahanovitch U*, et al.* Recruitment of Gβγ controls the basal activity of G-protein coupled inwardly rectifying potassium (GIRK) channels: crucial role of distal C terminus of GIRK1. *J Physiol* **592**, 5373–5390 (2014).

11. Berlin S*, et al.* Gα_i and G$\beta\gamma$ jointly regulate the conformations of a G$\beta\gamma$ effector, the neuronal G protein-activated K^+ channel (GIRK). *J Biol Chem* **285**, 6179–6185 (2010).

12. Abramson J*, et al.* Accurate structure prediction of biomolecular interactions with AlphaFold 3. *Nature* **630**, 493–500 (2024).

13. Qi Y, Ingólfsson HI, Cheng X, Lee J, Marrink SJ, Im W. CHARMM-GUI martini maker for coarse-grained simulations with the martini force field. *J Chem Theory Comput* **11**, 4486–4494 (2015).

14. Abraham MJ*, et al.* GROMACS: High performance molecular simulations through multi-level parallelism from laptops to supercomputers. *SoftwareX* **1-2**, 19–25 (2015).

15. Periole X, Cavalli M, Marrink SJ, Ceruso MA. Combining an elastic network with a coarse-grained molecular force field: Structure, dynamics, and intermolecular recognition. *J Chem Theory Comput* **5**, 2531–2543 (2009).

16. Yesylevskyy SO, Schäfer LV, Sengupta D, Marrink SJ. Polarizable water model for the coarse-grained MARTINI force field. *PLoS Comput Biol* **6**, e1000810 (2010).

17. Atsmon-Raz Y, Tieleman DP. Parameterization of palmitoylated cysteine, farnesylated cysteine, geranylgeranylated cysteine, and myristoylated glycine for the martini force field. *J Phys Chem B* **121**, 11132–11143 (2017).

18. Bussi G, Donadio D, Parrinello M. Canonical sampling through velocity rescaling. *J Chem Phys* **126**, 014101 (2007).

19. Parrinello M, Rahman A. Polymorphic transitions in single crystals: A new molecular dynamics method. *J Appl Phys* **52**, 7182–7190 (1981).

20. Michaud-Agrawal N, Denning EJ, Woolf TB, Beckstein O. MDAnalysis: a toolkit for the analysis of molecular dynamics simulations. *J Comput Chem* **32**, 2319–2327 (2011).

21. Jo S, Kim T, Iyer VG, Im W. CHARMM-GUI: a web-based graphical user interface for CHARMM. *J Comput Chem* **29**, 1859–1865 (2008).

22. Maier JA, Martinez C, Kasavajhala K, Wickstrom L, Hauser KE, Simmerling C. ff14SB: Improving the accuracy of protein side chain and backbone parameters from ff99SB. *J Chem Theory Comput* **11**, 3696–3713 (2015).

23. Peleg S, Varon D, Ivanina T, Dessauer CW, Dascal N. Gα_i controls the gating of the G-protein-activated K^+ channel, GIRK. *Neuron* **33**, 87–99 (2002).

24. Sarvazyan NA, Lim WK, Neubig RR. Fluorescence analysis of receptor-G protein interactions in cell membranes. *Biochemistry* **41**, 12858–12867 (2002).

25. Sawai T, Hirakawa T, Yamada K, Nishizawa Y. Interaction between Pleckstrin homology domains and G protein $\beta\gamma$ subunits: analyses of kinetic parameters by a biosensor-based method. *Biol Pharm Bull* **22**, 229–233 (1999).

26. Zheng S, Abreu N, Levitz J, Kruse AC. Structural basis for KCTD-mediated rapid desensitization of GABAB signalling. *Nature* **567**, 127–131 (2019).

27. De Waard M, Liu H, Walker D, Scott VE, Gurnett CA, Campbell KP. Direct binding of G-protein βγ complex to voltage-dependent calcium channels. *Nature* **385**, 446–450 (1997).

28. Savage JR, McLaughlin JN, Skiba NP, Hamm HE, Willardson BM. Functional roles of the two domains of phosducin and phosducin-like protein. *J Biol Chem* **275**, 30399–30407 (2000).

29. Carman CV*, et al.* Mutational analysis of Gbg and phospholipid interaction with G protein-coupled receptor kinase 2. *J Biol Chem* **275**, 10443–10452 (2000).

30. Runnels LW, Scarlata SF. Determination of the affinities between heterotrimeric G protein subunits and their phospholipase C-β effectors. *Biochemistry* **38**, 1488–1496 (1999).

31. Krapivinsky G, Krapivinsky L, Wickman K, Clapham DE. Gβγ binds directly to the G protein-gated K^+ channel, I_{KACh}. *J Biol Chem* **270**, 29059–29062 (1995).

32. Krapivinsky G, Kennedy ME, Nemec J, Medina I, Krapivinsky L, Clapham DE. Gβ binding to GIRK4 subunit is critical for G protein-gated K^+ channel activation. *J Biol Chem* **273**, 16946–16952 (1998).

33. Doupnik CA, Dessauer CW, Slepak VZ, Gilman AG, Davidson N, Lester HA. Time resolved kinetics of direct Gb_1g_2 interactions with the carboxyl terminus of Kir3.4 inward rectifier K^+ channel subunits. *Neuropharmacol* **35**, 923–931 (1996).

34. Yokogawa M, Osawa M, Takeuchi K, Mase Y, Shimada I. NMR analyses of the Gβγ binding and conformational rearrangements of the cytoplasmic pore of G protein-activated inwardly rectifying potassium channel 1 (GIRK1). *J Biol Chem* **286**, 2215–2223 (2011).

35. Wang W, Touhara KK, Weir K, Bean BP, MacKinnon R. Cooperative regulation by G proteins and Na^+ of neuronal GIRK2 K^+ channels. *Elife* **5**, (2016).

36. Jelacic TM, Sims SM, Clapham DE. Functional expression and characterization of G-protein-gated inwardly rectifying K+ channels containing GIRK3. *J Membr Biol* **169**, 123–129 (1999).

37. Albsoul-Younes AM*, et al.* Interaction sites of the G protein β subunit with brain G protein-coupled inward rectifier K^+ channel. *J Biol Chem* **276**, 12712–12717 (2001).

38. Luchian T*, et al.* A C-terminal peptide of the GIRK1 subunit directly blocks the G protein-activated K^+ channel (GIRK) expressed in *Xenopus* oocytes. *J Physiol* **505 (Pt 1)**, 13–22 (1997).

39. Schreibmayer W*, et al.* Inhibition of an inwardly rectifying K channel by G-protein α-subunits. *Nature* **380**, 624–627 (1996).

40. Ito H*, et al.* On the mechanism of G protein bg subunit activation of the muscarinic K^+ channel in guinea pig atrial cell membrane. Comparison with the ATP-sensitive K^+ channel. *J Gen Physiol* **99**, 961–983 (1992).

41. Rishal I, Porozov Y, Yakubovich D, Varon D, Dascal N. Gβγ-dependent and Gβγ-independent basal activity of G protein-activated K^+ channels. *J Biol Chem* **280**, 16685–16694 (2005).

42. Rubinstein M*, et al.* Divergent regulation of GIRK1 and GIRK2 subunits of the neuronal G protein gated K^+ channel by $G\alpha_i$GDP and Gβγ. *J Physiol* **587**, 3473–3491 (2009).

43. Berlin S*, et al.* Two distinct aspects of coupling between Gαi protein and G protein-activated K^+ channel (GIRK) revealed by fluorescently labeled $G\alpha_{i3}$ protein subunits. *J Biol Chem* **286**, 33223–33235 (2011).
