# Supplementary material for: Live-cell quantitative monitoring reveals distinct, high-affinity Gβγ regulations of GIRK2 and GIRK1/2 channels
Source: Nat Commun. 2025 Nov 24;16:11607. doi: 10.1038/s41467-025-66730-8 (PMC12748987; doi:10.1038/s41467-025-66730-8)
Supplement: Supplementary file 2 — Description of Additional Supplementary Files [file 41467_2025_66730_MOESM2_ESM.pdf]

Title: Supplementary Data 1

Description: MD simulation analysis details. This Table provides the details of analysis of MD simulations of the interaction of Gγ' prenylated tail ($G\gamma_{prenyl}$) with G1NC, $G1NC_{trunc}$, G2NC and $G2NC_{truc}$, and of G1NT-G1dCT interactions. It contains 6 sub-tables:

Sub-Table 1: G1NC - Prenylation Tail and NT-dCT binding

Table 2: Truncated G1NC - Prenylation Tail binding

Sub-Table 3: G2NC - Prenylation Tail binding

Sub-Table 4: Truncated G2NC - Prenylation Tail binding

Sub-Table 5: G1NC with Membrane - Prenylation Tail binding

Sub-Table 6: G1NC - all-atom simulations - Prenylation Tail binding

Title: Supplementary Data 2

Description: Primers using for DNA cloning. Oligonucleotides used as primers in the creation of DNA constructs and mutations described in the paper.
